# An updated checklist of mosquito species (Diptera: Culicidae) from Madagascar

**DOI:** 10.1051/parasite/2016018

**Published:** 2016-04-21

**Authors:** Michaël Luciano Tantely, Gilbert Le Goff, Sébastien Boyer, Didier Fontenille

**Affiliations:** 1 Laboratoire d’Entomologie Médicale, Institut Pasteur de Madagascar Antananarivo 101 Madagascar; 2 IRD UMR MIVEGEC, 34394 Montpellier and IRD La Réunion-GIP CYROI 97490 Sainte Clotilde La Réunion France; 3 IRD UMR MIVEGEC, 34394 Montpellier and Institut Pasteur du Cambodge 5 BP 983, Blvd. Monivong 12201 Phnom Penh Cambodia

**Keywords:** Mosquitoes, Taxonomy, Biology, Vectors, Madagascar

## Abstract

An updated checklist of 235 mosquito species from Madagascar is presented. The number of species has increased considerably compared to previous checklists, particularly the last published in 2003 (178 species). This annotated checklist provides concise information on endemism, taxonomic position, developmental stages, larval habitats, distribution, behavior, and vector-borne diseases potentially transmitted. The 235 species belong to 14 genera: *Aedeomyia* (3 species), *Aedes* (35 species), *Anopheles* (26 species), *Coquillettidia* (3 species), *Culex* (at least 50 species), *Eretmapodites* (4 species), *Ficalbia* (2 species), *Hodgesia* (at least one species), *Lutzia* (one species), *Mansonia* (2 species), *Mimomyia* (22 species), *Orthopodomyia* (8 species), *Toxorhynchites* (6 species), and *Uranotaenia* (73 species). Due to non-deciphered species complexes, several species remain undescribed. The main remarkable characteristic of Malagasy mosquito fauna is the high biodiversity with 138 endemic species (59%). Presence and abundance of species, and their association, in a given location could be a bio-indicator of environmental particularities such as urban, rural, forested, deforested, and mountainous habitats. Finally, taking into account that Malagasy culicidian fauna includes 64 species (27%) with a known medical or veterinary interest in the world, knowledge of their biology and host preference summarized in this paper improves understanding of their involvement in pathogen transmission in Madagascar.

## Abstract (fr)

Une liste mise à jour des 235 espèces de moustiques de Madagascar est présentée. Le nombre d’espèces a considérablement augmenté par rapport à celui donné dans les listes précédentes, en particulier la dernière, publiée en 2003 (178 espèces). Cette liste fournit des informations concises sur l’endémisme, la position taxonomique, les stades de développement, les gîtes larvaires, la distribution, le comportement des moustiques, et aussi sur les maladies potentiellement transmises par les moustiques vecteurs. Ces 235 espèces sont réparties dans 14 genres : *Aedeomyia* (3 espèces), *Aedes* (35 espèces), *Anopheles* (26 espèces), *Coquillettidia* (3 espèces), *Culex* (au moins 50 espèces), *Eretmapodites* (4 espèces), *Ficalbia* (2 espèces), *Hodgesia* (au moins une espèce), *Lutzia* (une espèce), *Mansonia* (2 espèces), *Mimomyia* (22 espèces), *Orthopodomyia* (8 espèces), *Toxorhynchites* (6 espèces), *Uranotaenia* (73 espèces). En raison de complexes d’espèces non résolus, plusieurs espèces sont toujours non décrites. La principale caractéristique remarquable de la faune malgache de moustiques est la grande biodiversité avec 138 espèces endémiques (59 %). La présence et l’abondance des espèces, et leur association dans un endroit donné, pourrait être un bio-indicateur des particularités environnementales telles que les habitats urbains, ruraux, forestiers, déboisés et montagneux. Enfin, compte tenu que la faune malgache de moustiques comprend 64 espèces (27 %) ayant un intérêt médical ou vétérinaire connu dans le monde, la connaissance de leur biologie et de leur préférence d’hôtes résumée dans le présent document permet de comprendre leur implication dans la transmission d’agents pathogènes à Madagascar.

## 1. Introduction

The first information about Malagasy mosquitoes dated from the second half of the 19th century, when the presence of *Aedes aegypti* (as *Culex insatiabilis*) (Linnaeus) and *Culex quinquefasciatus* Say (as *C. anxifer*) was recorded by Bigot (1859) [12]. The first description of a Malagasy mosquito species was made by a pioneer of tropical medicine, the parasitologist Alphonse Laveran, in 1900 and involved a new *Anopheles* (*An. coustani* Laveran) [137]. In 1920, Enderlein [81] and Edwards [75] were the first to report mosquito collections from Madagascar and the Mascareignes Islands in the Indian Ocean [75]. This observation highlights that knowledge about Malagasy Culicidae fauna was closely related to human health, like during health campaigns during World War II [53, 54, 228, 229] and then in studies by the *Institut de Recherche Scientifique de Madagascar* (IRSM) and *Institut Pasteur de Madagascar* (IPM). These institutes were responsible for further research on the mosquitoes of Madagascar in relation to malaria, filariasis, and arbovirus control programs. A large number of species were described by Doucet [63–68], Grjebine [103, 105–107], Brunhes [20–26], Brunhes and collaborators [28–31], Ravaonjanahary [183, 184], and Rodhain and Boutonnier [192, 193]. Grjebine presented a monograph of 26 *Anopheles* species [108], Ravaonjanahary studied the biogeography of the 23 *Aedes* species [182], Fontenille published the first checklist which included 177 Malagasy species [85], and Brunhes & Hervy published a monograph of *Orthopodomyia* species of the Ethiopian region [27]. The last revised checklist was published in 2003 and included 178 mosquito species [70]. Considering the medical and nuisance impact of the genera *Aedes*, *Anopheles*, and *Culex* in pathogen transmission, the checklists included more species belonging to these genera [70, 85].

Species belonging to the genera *Uranotaenia* [51], *Toxorhynchites* [190], subgenus *Aedes* (*Neomelaniconion*) [143] and *Aedeomyia* [33] were recently described.

The annotated checklist was developed with the aim of updating the list of Malagasy mosquito species, to eliminate species erroneously mentioned in Internet reference such as http://mosquito-taxonomic-inventory.info/ [119], *Arthropodes d’intérêt médical* (Arim: http://www.arim.ird.fr/) [5]; and the Walter Reed Biosystematics Unit (WRBU) at the Smithsonian Institution WRBU: http://wrbu.si.edu/ [244], and to provide more knowledge on their systematics, bioecology, vectorial capacity, distribution, and vector-borne disease status.

This list was compiled using our own observations, and Internet and bibliographic references. The Culicidae mosquito fauna includes 235 species within 14 genera. The present taxon identifications are based on formally recognized genera, and subgenera. Their abbreviations follow taxonomic nomenclature from *A Catalog of the Mosquitoes of the World* [132], its supplements [131, 235, 236] and Reinert [188, 189], and Wilkerson et al. [241] for the names of tribe Aedini. The author is given at first mention of a species.

Each species can be cited as follows: genus (subgenus) species, author(s) and date of first description, new name according to Wilkerson et al. [241], author(s) and date of first mention in Madagascar, endemism, development stages, larval habitats, distribution, trophic behavior, and vector-borne diseases potentially transmitted. The relationship between the species distribution and the importance of Malagasy biodiversity has been discussed, raising questions about the mosquito’s evolution and differentiation.

## 2. Presentation of Malagasy mosquitoes

### 2.1 Genus *Anopheles* Meigen, 1818

The genus *Anopheles* is subdivided into eight subgenera. The subgenera *Anopheles* and *Cellia* are present in Madagascar. The subgenus *Anopheles* is represented by more than 183 species in the world [119]. In Madagascar, three species are present and they belong to the Myzorhynchus Series. One is an endemic species. The subgenus *Cellia* is represented by 224 species in the world. In Madagascar, 23 species occur. Ten are endemic, and one species (*Anopheles mascarensis* de Meillon) is present in Madagascar and in the Comoros Islands. *Anopheles* (*Ano.*) *obscurus* (Grünberg), *An.* (*Cel.*) *argenteolobatus* (Gough), *An.* (*Cel.*) *christyi* (Newstead & Carter), *An.* (*Cel.*) *confusus* Evans & Leeson, *An.* (*Cel.*) *marshallii* (Theobald), *An.* (*Cel.*) *nili* (Theobald), and *An.* (*Cel.*) *theileri* Edwards are absent from Madagascar (this study, [5]) but were ranked by WRBU among the mosquito species found on the island [244]. Three names of doubtful validity (*An. arnoulti*, *An. courdurieri*, and *An. fuscicolor soalalaensis*), regarded as *nomen dubium* by Brunhes et al. [32], are not listed in this document.

#### 2.1.1 Subgenus *Anopheles* Meigen, 1818

Myzorhynchus Series [78]

Group Coustani [186]

- ***Anopheles* (*Anopheles*) *coustani* Laveran, 1900** [137]

Laveran, 1900 [137]

In Madagascar, larval habitats are cattle hoof prints [63], ponds, swamps [103], rivers, streams, canals, rice fields [67], lakes, rock holes, flushing holes [140], and pools of brackish water [108]. Occurs in all biogeographic domains [85, 108]. Zoo-anthropophilic species, involved in transmission of *Plasmodium falciparum* and *P. vivax* [169], *Wuchereria bancrofti* [20], *Setaria* sp. [23], endemic Périnet virus (PERV) [85], Rift Valley fever virus (RVFV) [181], and West Nile virus (WNV) [152]. Babanki virus (BABV) was found in a mixed batch of mosquito species, including *An. coustani*, collected in Périnet [85]. In Africa, involved in transmission of Zika virus (ZIKV) [56]. Oocysts and sporozoite stage of *Plasmodium*, causing human malaria, were reported [239].

- ***Anopheles* (*Anopheles*) *fuscicolor* van Someren, 1947** [228]

van Someren, 1947 [228]

Endemic. Eggs undescribed. Larval habitats are ponds, streams, rice fields, irrigation canals, marshes, ponds, lakes, rivers, water bodies, and lagoons [108]. Occurs in all Malagasy biogeographic domains [108]. BABV, RVFV, and PERV were isolated from a mixed batch of mosquito species, including *Anopheles fuscicolor* [85]. *Wuchereria bancrofti* stage III found [23].

- ***Anopheles* (*Anopheles*) *tenebrosus* Dönitz, 1902** [61]

Grjebine, 1956 [105]

In Madagascar, larval habitats are rice fields, ponds, swamps, and streams. Occurs in the Sambirano (Nosy Be), northern, central, and western biogeographic domains [108]. Not involved in disease transmission in Madagascar, but involved in transmission of human malaria in Africa [3].

#### 2.1.2 Subgenus *Cellia* Theobald, 1902

Pyretophorus Series [78]

Gambiae Complex

**Figure 1.**
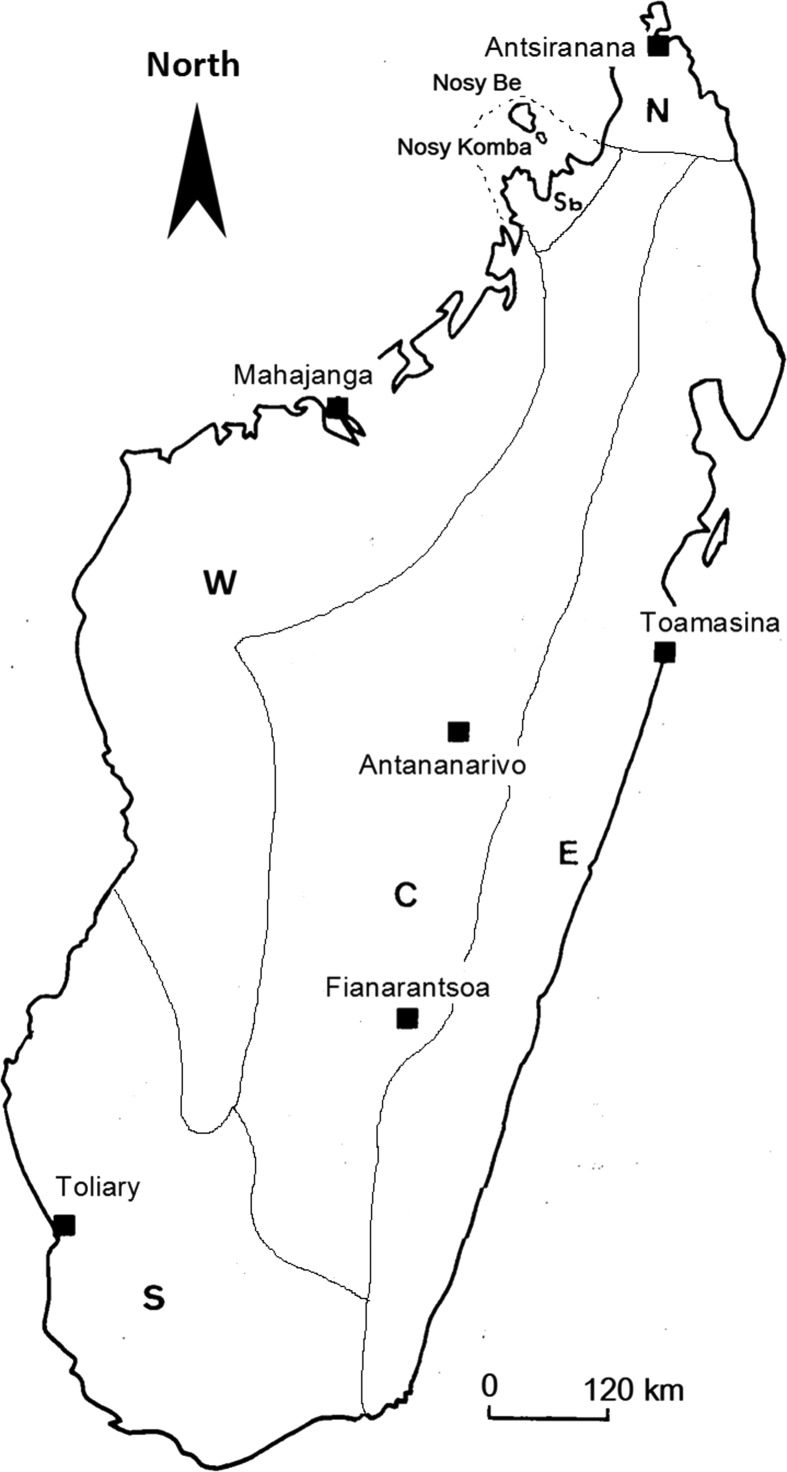
Map showing the biogeographic domains of Madagascar. **N:** northern domain; **W:** western domain; **S:** southern domain; **E:** eastern domain; **C:** central domain and Sb: Sambirano area [46].

This complex is represented by eight species in Africa. Three of them occur in Madagascar: *Anopheles gambiae* Giles, *An. arabiensis* Patton, and *An. merus* Dönitz. All developmental stages of these three species have been described. *Anopheles gambiae* and *An. arabiensis* grow in freshwater breeding sites. *Anopheles merus* grows in brackish water and occurs in the southern and western biogeographic domains of Madagascar [146]. These three species are vectors of *Plasmodium* sp. [91, 146]. They are also involved in transmission of *Wuchereria bancrofti* [20]. In Africa, these three species were found naturally infected with Mengo virus (MgV) [85], Ganjam virus (GANV) [101], Tataguine virus (TATV), Ilesha virus (ILEV) [45], O’nyong-nyong virus (ONNV) [231], and Bwamba virus (BWAV) [47] (Fig. 1).

- ***Anopheles* (*Cellia*) *arabiensis* Patton, 1905** [172]

Chauvet & Déjardin, 1968 [37]

In Madagascar, it has karyotypes similar to those of east Africa [179], shows high and low degrees of zoophilic and anthropophilic behavior, respectively [179], and occurs in all biogeographic domains [36, 146].

- ***Anopheles* (*Cellia*) *gambiae* Giles, 1902** [97]

Chauvet & Déjardin, 1968 [37]

On the African mainland, five chromosomal forms, partially panmictic [43, 44] and two DNA ribosomal molecular forms, were described [83]. These molecular forms M and S have recently been elevated to the status of species and are named respectively: *Anopheles coluzzii* Coetzee & Wilkerson and *An*. *gambiae* s.s. [42]. In Madagascar, *Anopheles gambiae* has molecular forms similar to those of east Africa [142, 146, 209] and the species identified is *Anopheles gambiae* s.s. This species is characterized by a high degree of anthropophilic behavior [179] with notable exceptions [69]. In Madagascar, present in all biogeographic domains [36, 146, 209].

- ***Anopheles* (*Cellia*) *merus* Dönitz, 1902**

Chauvet, 1968 [36]

Larval habitats are brackish water in the Mangatsa area, and crab holes in Betsiboka estuary (in the Mahajanga region), in the western and southern domains, and in the extreme south of Madagascar [146, 170]. Major vector of human *Plasmodium* in local scale [146].

Myzomyia Series (Christophers, 1924) [39]

- ***Anopheles* (*Cellia*) *brunnipes* (Theobald, 1910)** [220]

Doucet, 1951

Eggs undescribed. In Madagascar, larval habitats are ponds, along riverbanks, streams, road drains, ditches, nurseries, and rice fields [108]. Occurs in the western, central, and eastern domains [108]. WNV was isolated from specimens caught in Manambo area [85]. In the Ethiopian region, it has a wide distribution, and oocysts and sporozoites of *Plasmodium* causing human malaria were reported for this species [239].

- ***Anopheles* (*Cellia*) *flavicosta* Edwards, 1911** [72]

Coz, Grjebine & Hamon, 1960 [49]

Eggs undescribed. In Madagascar, larval habitats are ponds, rivers, streams, and rice fields [108]. Occurs in all biogeographic domains [85, 108]. In the Ethiopian region, it has a wide geographical distribution and is highly zoophilic, and occasionally feeds on humans [114]. Involved in transmission of *Wuchereria bancrofti* [16], *Plasmodium* sp., and Middelburg virus (MIDV) [1, 239].

Group Funestus (Harbach 2004) [116]

Subgroup Funestus (Gillies and de Meillon, 1968) [98]

- ***Anopheles* (*Cellia*) *funestus* Giles, 1900** [95]

Laveran, 1904 [138]

The Group Funestus includes 10 species. In Madagascar, only the species *Anopheles funestus* is present. Larval habitats are irrigation canals [67], lakes, ponds, pools, marshes, riverbanks, streams, irrigation drains, and rice fields [108]. Occurs in all Malagasy biogeographic domains [85, 108]. Anthropophilic species and major vector of malaria parasite [91] and involved in transmission of *Wuchereria bancrofti* and *Setaria* sp. [23]. In the Ethiopian region, widely distributed and involved in transmission of Pongola virus (PGAV) [47], ONNV, BWAV, Nyando virus (NDV) [151], Chikungunya virus (CHIKV), Wesselsbron virus (WSLV), Bozo virus (BOZOV), Akabane virus (AKAV), Tanga virus (TANV), TATV, and Orungo virus (ORUV) [1].

Cellia Series (Christophers, 1924) [39]

Group Squamosus Grjebine, 1966 [108]

- ***Anopheles* (*Cellia*) *cydippis* de Meillon, 1931** [52]

Doucet, 1951 [68]

Adults are morphologically similar to *Anopheles squamosus* Theobald. Its larval stages differ from those of *Anopheles squamosus*, in having simple external clypeal seta (3-C), plumose, or with few short or long branches. In Madagascar, larval habitats are lakes, ponds, marshes, riverbanks, streams, pools, water containing iron hydroxide, irrigation drains, tanks, cement basins [108], and watering holes [140]. Occurs in all biogeographic domains [85, 108]. Not involved in disease transmission.

- ***Anopheles* (*Cellia*) *squamosus* Theobald, 1901**

Laveran, 1904 [138]

Eggs undescribed. Its larval stages differ from those of *Anopheles cydippis*, in having a dendroid external clypeal seta (3-C) with one trunk divided into eight branches. In Madagascar, larval habitats are marshes [103], ponds, rice fields [111], irrigation drains, pools, brackish water pools, rivers, and lagoons [108]. Occurs in all biogeographic domains [85, 108]. Zoophilic species (cattle, sheep, and poultry) [210] and involved in transmission of RVFV [181], Andasibe virus (ANDV) [85], Bluetongue virus (BTV) [4], and *Wuchereria bancrofti* [23]. In Africa, zoophilic species [114] and involved in transmission of Birao virus (BIRV) [45] and BABV [1].

- ***Anopheles* (*Cellia*) *pharoensis* Theobald, 1901**

Ventrillon, 1905 [75]

In Madagascar, larval habitats are cattle hoof prints, grasslands [63], ponds, rice fields [67], lagoons (fresh water), drains, irrigation, nurseries, lakes, ponds, marshes, riverbanks, streams, and water containing iron hydroxide [108]. Occurs in all biogeographic domains (except the Sambirano area) [85, 108]. Not involved in disease transmission. In Africa, occurs in Ethiopian region, Egypt, and Eritrea. Essentially zoophilic (especially cattle) and may feed on birds [114]. Involved in transmission of BIRV [45], RVFV [147], Ngari virus (NGAV), Bangui virus (BGIV) [101], BABV, WSLV, Sanar virus (SANV) [1], *Wuchereria bancrofti* [16], and *Plasmodium* sp. (with oocysts and sporozoites) [239].

Neocellia Series (Christophers, 1924) [39]

- ***Anopheles* (*Cellia*) *maculipalpis* Giles, 1902** [97]

Monier, 1937 [167]

Eggs undescribed. In Madagascar, larval habitats are marshes, ponds, riverbanks, streams, wetlands, lakes, irrigation drains, tanks, cement tanks, tire tracks, cattle hoof prints, plant nurseries, and rice fields [108]. Occurs in all biogeographic domains [85, 108] and may be attracted to humans and livestock [85] (Luciano Tantely, unpublished observation). Involved in transmission of WNV [85]. In Africa, involved in transmission of *Wuchereria bancrofti* [16] and *Plasmodium* sp. (oocysts or sporozoites) [239].

- ***Anopheles* (*Cellia*) *rufipes* (Gough, 1910)** [102]

Wilson, 1947 [243]

In Madagascar, larval habitats are lakes, ponds, pools, marshes, wetlands, containing water with more or less iron hydroxide, streams, puddles, rock holes, tanks, cement tanks, nurseries, and rice fields [108]. Occurs in all Malagasy biogeographic domains [85, 108, 185]. May be attracted to humans [85] and livestock (Luciano Tantely, unpublished observation); but not involved in disease transmission. In Africa, zoophilic species (particularly to large mammals) [114] and involved in transmission of CHIKV [57], WSLV, and Gomoka virus (GOMV) [1] and *Plasmodium* sp. (oocysts or sporozoites) [239].

- ***Anopheles* (*Cellia*) *pretoriensis* (Theobald, 1903)** [216]

Grjebine, 1953 [103]

In Madagascar, larval habitats are rock holes [112], marshes, ponds, riverbanks, streams, puddles, irrigation drains, and rice fields [108]. Occurs in all Malagasy biogeographic domains [85, 108]. Anthropophilic species [108] and may be attracted to livestock (Luciano Tantely, unpublished observation), but not involved in disease transmission. In Africa, involved in transmission of WSLV and NGAV in Senegal [1], and *Plasmodium* sp. (oocysts or sporozoites) [239].

Series Neomyzomyia (Christophers, 1924) [39]

Group Mascarensis (Harbach, 1994) [116]

- ***Anopheles* (*Cellia*) *mascarensis* de Meillon, 1947** [54]

Endemic in Madagascar and Comoros archipelagos. Eggs undescribed. In Madagascar, it was confused with *An.* (*Cellia*) *marshallii* (African mainland species) [108]. Larval habitats are streams [103], lakes, ponds, pools, marshes, riverbanks, backwaters, irrigation drains, nurseries, brackish water, lagoons [108], wetland pools [18], and rice fields [191]. Occurs in Sambirano domain (Nosy Be, Nosy Komba) [90], and in all Malagasy biogeographic domains [85, 108]. May be attracted to livestock, humans, and poultry [141, 210]. Secondary or major vector of local importance for malaria [87, 141, 146, 154]. Found naturally infected with NGAV [85] and *Wuchereria bancrofti* [23].

Group Pauliani (Grjebine, 1966) [108]

- ***Anopheles* (*Cellia*) *grassei* Grjebine, 1953** [104]

Grjebine, 1953 [104]

Endemic. Only species belonging to the Grassei Group. Morphologically close to *Anopheles radama* de Meillon. Larval habitats are marshes, ponds, backwaters, coastal rivers, streams, bodies of water due to small dams, and tree holes [85]. Occurs in the eastern [108] and western domains [15, 170]. May be caught in human landing catches [85], but is not involved in transmission of vector-borne diseases.

- ***Anopheles* (*Cellia*) *grenieri* Grjebine, 1964** [107]

Grjebine 1964 [107]

Endemic. Only the larval stages were described [107]. Larval habitats are streams flowing through forest and harvested rice fields [108]. Occurs in the eastern wetland area [108] and not involved in disease transmission.

- ***Anopheles* (*Cellia*) *milloti* Grjebine & Lacan, 1953** [103]

Grjebine & Lacan, 1953 [103]

Endemic. Eggs undescribed. Larval habitats are holes, grassy ponds, lakes, ponds, riverbanks of streams, and lakes, water containing iron hydroxide, irrigation drains [108, 112], swamps, and marshland [18]. Occurs in all Malagasy biogeographic domains (except the southern domain) [108]. Adult biology unknown. Not involved in transmission of vector-borne diseases.

- ***Anopheles* (*Cellia*) *pauliani* Grjebine, 1953** [103]

Grjebine, 1953 [103]

Endemic. Eggs undescribed. Larval habitats are lakes, ponds, pools, marshes, ponds, rivers, streams, and rice fields [108]. Occurs in the Sambirano area (Nosy Komba) [90], and all Malagasy biogeographic domains [85, 108, 185], prefers to feed on domestic ruminants, but it may also feed on humans, birds, rodents, and lemurs [108]. RVFV and ANDV were found in a mixed batch of mosquito species, including *An. pauliani*, caught in Périnet [85]. Involved in transmission of WNV [152] and *Wuchereria bancrofti* [23].

- ***Anopheles* (*Cellia*) *radama* de Meillon, 1943** [53]

de Meillon, 1943 [53]

Endemic. Eggs undescribed. Morphologically close to *Anopheles grassei* Grjebine. Larval habitats are ponds [66], streams, bodies of water, rivers, and volcanic crater lakes [108]. Occurs in the Sambirano area (in Nosy Be, Nosy Komba) [90], the northern [85, 108], western [185], and eastern domains [66]. Not involved in transmission of vector-borne diseases.

Group Ranci (Grjebine, 1966) [108]

Subgroup Ranci (Grjebine, 1966) [108]

- ***Anopheles* (*Cellia*) *griveaudi* Grjebine, 1960** [106]

Grjebine, 1960 [106]

Endemic. Only the adult female was described [106] and is known only from a single specimen collected in 1956, by Griveaud, in a mercury-vapor lamp, in Ankaratra massif, in the central biogeographic domain [108].

- ***Anopheles* (*Cellia*) *ranci* Grjebine, 1953** [103]

Grjebine, 1953 [103]

Endemic. Eggs undescribed. Larval habitats are pools, riverbanks, streams, and rice fields [108]. Occurs in the northern and eastern biogeographic domains [85, 108]. Adult biology unknown. Not involved in transmission of vector-borne diseases.

Subgroup Roubaudi (Grjebine, 1966) [108]

- ***Anopheles* (*Cellia*) *lacani* Grjebine, 1953** [103]

Grjebine, 1953 [103]

Endemic. Adult male and eggs undescribed. Larval habitats are streams flowing through natural forest areas [108, 207]. Occurs in the Mandraka forest of the eastern wetland domain [108] and in the Anjozorobe-Angavo forest corridor, in the subhumid area of the central biogeographic domain [210]. Adult biology unknown. Not involved in transmission of vector-borne diseases.

- ***Anopheles* (*Cellia*) *notleyi* van Someren, 1949** [229]

van Someren, 1949 [229]

Endemic. Eggs undescribed. Larval habitats are streams flowing through forest areas [108]. Occurs only in Sakaramy area, Antsiranana province, in the northern domain [108] and in Farankaraina forest, near the Masoala National Park, in the eastern domain (Gilbert Le Goff, unpublished observation). Adult biology unknown. Not involved in transmission of vector-borne diseases.

- ***Anopheles* (*Cellia*) *roubaudi* Grjebine, 1953** [103]

Grjebine, 1953 [103]

Endemic. Eggs undescribed. Wing morphology similar to that of *Anopheles flavicosta* Edwards, *An. notleyi*, *An. lacani* [108]. Larval habitats are streams flowing through medium altitude forests (900 m asl), like Périnet (Gilbert Le Goff, unpublished observation). Occurs in the humid forest of the eastern domain [108]. Adult biology unknown. Not involved in transmission of vector-borne diseases.

### 2.2 Genus *Aedeomyia* Theobald, 1901 [213]

This genus is subdivided into two subgenera: *Aedeomyia* and *Lepiothauma*. It is represented by seven species distributed in Afrotropical, Australasian, Oriental, and Neotropical regions [33, 117]. Three species occur in Madagascar: two of them are Malagasy endemic species [33]. Larvae of this genus often breed in permanent water with abundant aquatic vegetation. Little is known about adult biology.

#### 2.2.1 Subgenus *Aedeomyia* Theobald, 1901

This subgenus is represented by six species in the world. Two species are endemic to Madagascar. Species of genus *Aedeomyia* has been documented to feed on birds [33].

- ***Aedeomyia* (*Aedeomyia*) *madagascarica* Brunhes, Boussès, & da Cunha Ramos, 2011** [33]

Brunhes, Boussès, & da Cunha Ramos, 2011 [33]

Endemic. Known only in the adult stages (male and female) [33]. Biology unknown. Collected from the type locality (forest of Ivoloina, Toamasina) [33] and in the western domain [15]. Ornithophilic around Lake Kinkony [211] and found naturally infected with WNV in Mitsinjo district [152].

- ***Aedeomyia* (*Aedeomyia*) *pauliani* Grjebine, 1953** [103]

Grjebine, 1953 [103]

Endemic. Collected only once, known only from the type locality (Lake Zanavorono, Ambila Lemaitso), and only from the larval stages collected from the banks of a lake in Ambila Lemaitso, on the Pangalanes Canal, in the eastern domain of Madagascar [22].

#### 2.2.2 Subgenus *Lepiothauma* Enderlein, 1923

This subgenus is monotypic.

- ***Aedeomyia* (*Lepiothauma*) *furfurea* (Enderlein, 1923)** [82]

Doucet, 1950 [65]

Eggs undescribed. Wide spatial distribution in Africa and Madagascar. In Madagascar, larvae were found breeding in muddy swamps, rice fields, ponds [67], crater lakes [108], and fishponds [33]. Collected for the first time by Paulian from Antsohihy of the western domain [65]; also found in central and eastern domains [33]. Not involved in disease transmission in Madagascar.

### 2.3 Genus *Aedes* Meigen, 1818

*Aedes* is the largest genus in the subfamily Culicinae with 930 species [241]. Thirty-eight subgenera were reported by Knight and Stone in 1977 [132]. This genus was recently subdivided into 74 subgenera, which was restored to its status prior to the year 2000 [241]. According to Wilkerson et al. [241], 12 subgenera are present in Madagascar. These subgenera are represented by 35 species, 18 species are endemic to Madagascar, and two species are found in Madagascar and in the Comoros archipelago.

#### 2.3.1 Subgenus *Aedimorphus* Theobald, 1903

In Madagascar, this subgenus includes eight species, three are endemic. This list and Arim [5] did not include *Aedes* (*Aedimorphus*) *ovazzai* that may be erroneously reported to be present on the island by WRBU [244]. In Africa, species were involved in transmission of RVFV [93, 147].

- ***Aedes* (*Aedimorphus*) *albodorsalis* Fontenille & Brunhes, 1984**

Fontenille & Brunhes, 1984 [86]

Endemic. Only the adult female has been described [86]. Found in the eastern [86] and western bioclimatic domains [15]. Anthropophilic, diurnal, seems closely related to north-eastern forest areas [86]. Not involved in disease transmission in Madagascar.

- ***Aedes* (*Aedimorphus*) *durbanensis* (Theobald, 1903)**

Ravaonjanahary, 1978 [182]

Pupal stages undescribed. In Madagascar, larval habitats are ponds, grassy bottom-land, and ditches [182]. Occurs in southern and western Malagasy domains [85, 170]. No medical and veterinary importance in Madagascar. However, was found naturally infected with RVFV in Kenya [168].

- ***Aedes* (*Aedimorphus*) *domesticus* (Theobald, 1901)**

Doucet, 1951 [67]

Only Malagasy species belonging to the Group Domesticus [80]. Larval stages morphologically close to *Aedes leptolabis* Edwards which is absent from Madagascar [230]. In Madagascar, larval habitats are puddles near the sea [67]. Presence reported in Madagascar by Doucet [67] in Vangaindrano (eastern domain) but never confirmed. In Africa, involved in transmission of Bunyamwera virus (BUNV) in Cameroon [1].

- ***Aedes* (*Aedimorphus*) *fowleri* (de Charmoy, 1908)**

Doucet, 1950 [65]

Egg and pupal stages undescribed. In Madagascar, larval habitats are rice fields, ponds, rock holes, rock crevices, and boreholes [103]. Occurs in all Malagasy biogeographic domains with the exception of the north [65, 85, 170]. Not involved in disease transmission in Madagascar. In Africa, zoophilic and would rather feed on livestock and birds [114]. Involved in transmission of Bagaza virus (BAGV) [58], ZIKV, Kedougou virus (KEDV), Simbu viruses (SIMV), PGAV, RVFV [1], and *Setaria* sp. [16].

- ***Aedes* (*Aedimorphus*) *dalzieli* (Theobald, 1910)**

Ravaonjanahary, 1978 [182]

Egg and pupal stages undescribed. Only Malagasy species belonging to the Group Abnormalis [80]. In Madagascar, larval habitats are rain puddles [182]. Occurs in southern and western domains [182]. Not involved in the transmission of disease in Madagascar. In Africa, zoophilic and may feed on cattle, birds, and bats [114]. Found naturally infected with RVFV [164], Dengue 2 virus (DENV-2) [223], CHIKV, BABV, MIDV, Ndumu virus (NDUV), BAGV, WSLV, WNV, Bouboui virus (BOUV), KEDV, BUNV, Shokwe virus (SHOV), NGAV, SIMV, PGAV [1], ZIKV [56], and Nematoda (undetermined species) [16].

- ***Aedes* (*Aedimorphus*) *masoalensis* Fontenille & Brunhes, 1984**

Fontenille & Brunhes, 1984 [86]

Endemic. Only the adult female was described [86]. Occurs in the eastern Malagasy domain. Diurnal and anthropophilic [86]. MgV was isolated from specimens caught in Toamasina [85].

- ***Aedes* (*Aedimorphus*) *mathioti* Fontenille & Brunhes, 1984**

Fontenille & Brunhes, 1984 [86]

Endemic. Only adult female was described [86]. Rare, occurs in the lowland forest of the eastern Malagasy domain (Onive river valley and region of Soanierana-Ivongo). Diurnal and anthropophilic [86]. Not involved in disease transmission.

- ***Aedes* (*Aedimorphus*) *natronius* Edwards, 1932**

Arim, 1959 [5]

Egg and pupal stages undescribed. No literature has reported its presence in Madagascar. However, this species occurs in Toliara, southern domain, as shown on the labels of specimens stored at the *Institut de Recherche pour le Développement* (IRD) of Montpellier [5]. Biology unknown. Not involved in disease transmission in Madagascar. In Africa, arboreal and crepuscular species [113] and involved in transmission of Uganda S virus (UGSV) [59].

#### 2.3.2 Subgenus *Catageiomyia* Theobald, 1903

Only one species belonging to the subgenus *Catageiomyia* is in Madagascar.

- ***Aedes* (*Catageiomyia*) *argenteopunctatus* (Theobald, 1901)**

Doucet, 1951 [67]

Eggs undescribed. In Madagascar, Group Argenteopunctatus is represented only by *Aedes argenteopunctatus* [80] which occurs in eastern and central domains [85, 182]. Known larval habitats in Madagascar: small pools of water near the ocean, in the Vangaindrano area [67]. Anthropophilic. Potential vector of Dakar Bat virus (DBV) [85]. In Africa, zoophilic and prefers to feed on domestic ruminants, but may also feed on humans [114]. Involved in transmission of Semliki Forest virus (SFV) [159], Nkolbisson virus (NKOV) [197], SHOV, MIDV [47], DENV-2 [223], CHIKV [57], WSLV, BUNV, PGAV, GOMV, NGAV [1], and Nematoda (undetermined species) [16].

#### 2.3.3 Subgenus *Coetzeemyia* Huang, Mathis, & Wilkerson, 2010

This subgenus was recently created by Huang et al. [126] and is monotypic.

- ***Aedes* (*Coetzeemyia*) *fryeri* (Theobald, 1912)**

Edwards, 1920 [75]

Egg and pupal stages undescribed. Subgenus was changed on several occasions. Presence reported in Madagascar by Edwards in 1920 [75]. Adult stage morphologically close to *Aedes dufouri* Hamon which is endemic to La Réunion and likely absent from Madagascar. In Madagascar, larval habits are rock holes [144] and its biology seems to be related to the presence of mangrove [182]. Occurs in the western and southern biogeographic domains [85]. Caught in abundance in human landing catches in the Morondava region. No medical and veterinary importance in Madagascar [85], but found naturally infected with Spondweni virus (SPOV) in a mixed-species batch of mosquitoes in Mozambique [161].

#### 2.3.4 Subgenus *Diceromyia* Theobald, 1911

In Madagascar, this subgenus is represented by five endemic species.

- ***Aedes* (*Diceromyia*) *coulangesi* Rodhain & Boutonnier, 1982**

Rodhain & Boutonnier, 1982 [192]

Endemic. Only the adult female and male were described to date [192]. Occurs in the Marovoay region (dry forest of Ampijoroa), in the western biogeographic domain [192]. Presence reported in the Montagne d’Ambre (northern biogeographic domain) and in Amboasary regions (southern biogeographic domain) [85]. Anthropophilic but no medical and veterinary importance in Madagascar [85].

- ***Aedes* (*Diceromyia*) *grassei* Doucet, 1951**

Doucet, 1951 [66]

Endemic. Egg, larval and pupal stages undescribed. Adult male morphologically close to *Ae. sylvaticus* [26]. Seems to occur only in the primary mountain forest of Moramanga and Périnet, in the eastern biogeographic domain [182]. No medical and veterinary importance in Madagascar.

- ***Aedes* (*Diceromyia*) *madagascarensis* van Someren, 1949**

van Someren, 1949, endemic [229]

Endemic. Adult male and female described [192]. Presence reported in all Malagasy biogeographic domains (except the southern domain) [85]. Diurnal and anthropophilic species and found naturally infected with WNV [85].

- ***Aedes* (*Diceromyia*) *sylvaticus* Brunhes, 1982**

Brunhes, 1982 [26]

Endemic. Only the adult male described. Morphologically close to *Aedes grassei* [26]. Collected only once and known only from the type locality (Ambohitranana forest, Masoala peninsula forest, eastern biogeographic domain) [26]. No medical and veterinary importance in Madagascar.

- ***Aedes* (*Diceromyia*) *tiptoni* Grjebine, 1953**

Grjebine, 1953 [103]

Endemic. Larval habitats are tree holes (mango tree, kapok tree, coconut tree, palm tree (*Medemia nobilis*) [182]. Occurs in all biogeographic domains of Madagascar [85, 170, 182]. Diurnal, anthropophilic, exophilic, and exophagic species [182]. May be attracted to domestic ruminants (goats and cattle) (Luciano Tantely, unpublished observation). No medical and veterinary importance in Madagascar.

#### 2.3.5 Subgenus *Fredwardsius* Reinert, 2000

This subgenus includes only a single species.

- ***Aedes* (*Fredwardsius*) *vittatus* (Bigot, 1861)**

Doucet, 1951 [67]

Eggs undescribed. Formerly classified into the subgenus *Stegomyia* [201]. In Madagascar, larval habitats are especially rock holes [67] and rice fields [103]. Occurs in the Sambirano area (Nosy Be, Nosy Komba), western, central [85], eastern [67], and southern domains [182]. In Madagascar, human landing catches seem to be a productive method for collecting *Aedes vittatus*. No medical and veterinary importance in Madagascar. In Africa, zoophilic [114] and involved in the transmission of DENV-2 [223], CHIKV [57], ZIKV, Yellow Fever virus (YFV), WSLV, Saboya virus (SABV), NGAV, SIMV, PGAV, GOMV [1], and Sindbis virus (SINV) [47].

#### 2.3.6 Subgenus *Mucidus* Theobald, 1901

This subgenus includes 11 species. The larvae of this subgenus are voracious predators of mosquito-associated species. Two species are present in Madagascar.

- ***Aedes* (*Mucidus*) *scatophagoides* (Theobald, 1901)**

Brunhes, 1968 [182]

Eggs undescribed. Presence reported for the first time in Madagascar by Brunhes in 1968 [182]. Larval habitats are warm temporary pools exposed to sunlight [182]. Occurs in the southern domain, particularly in the Antanimena area, in the semi-arid Androy region [182]. No medical and veterinary importance in Madagascar.

- ***Aedes* (*Mucidus*) *mucidus* (Karsch, 1887)**

Grjebine, 1955 [182]

Eggs undescribed. For the first time Grjebine (1955) reported the presence of *Ae. mucidus* in Périnet, eastern domain of Madagascar [182]. On the island, larval habitats are still unknown. No medical and veterinary importance in Madagascar.

#### 2.3.7 Subgenus *Neomelaniconion* Newstead, 1907

This subgenus includes 28 species. Six species of *Neomelaniconion* are present in Madagascar. Five species of them are endemic and were described from specimens formerly called *Ae* (*Neo.*) *palpalis* that is absent from Madagascar [5, 143], but ranked among the mosquito species found on the island by WRBU [244]. Vertical transmission of RVFV was described in species belonging to this subgenus [147].

- ***Aedes* (*Neomelaniconion*) *albiradius* (Le Goff, Boussès, & Brunhes, 2007)**

Le Goff, Boussès, and Brunhes, 2007 [143]

Endemic. Only the adult female was described [143]. Sequence variations of the ribosomal DNA ITS2 consistent with morphological observations, indicating that this species belongs to the Group Sylvaticum [130]. Occurs in forest areas of the western (forest station Ampijoroa), central (forest relic near Anjiro), and south (degraded forest near Mahabo) regions [143]. No medical and veterinary importance in Madagascar.

- ***Aedes* (*Neomelaniconion*) *belleci* (Le Goff, Boussès, & Brunhes, 2007)**

Le Goff, Boussès, & Brunhes, 2007 [143]

Endemic. Eggs undescribed. Variations in ribosomal DNA ITS2 sequences consistent with morphological observations, indicating that this species belongs to the Group Circumluteolus [130]. No specific differentiation at the molecular level obtained between *Ae*. *belleci* and *Ae*. *nigropterum*. In Madagascar, larval habitats are temporary ponds characterized by being slightly brownish in color and full of dead leaves and located in forest areas of medium altitude (1000 m asl), near Ranomafana, in the eastern biogeographic domain [143]. Adult biology is unknown because adults were obtained only from larval rearing. No medical and veterinary importance in Madagascar.

- ***Aedes* (*Neomelaniconion*) *circumluteolum* (Theobald, 1908)**

Hamon, 1959 [182]

Pupal stages undescribed. Presence reported in Madagascar in the southern domain by Hamon in 1959 [182] and confirmed by molecular study which indicated that Malagasy and South African specimens share a common origin [130]. In Madagascar, larval habitats are temporary pools and puddles [28]. Occurs in coastal areas, in Nosy Be, in western, eastern, and central biogeographic domains [85]. Never captured in the semi-arid bioclimatic domains of south and south-west Madagascar [85]. Diurnal and anthropophilic species in forested areas [143]. Found naturally infected with WNV in Ampijoroa and involved in transmission or dissemination of this virus in Madagascar [85]. In Africa, prefers to feed on cattle and may feed occasionally on humans [114, 182]. Involved in transmission of SIMV [45], WNV [127], SPOV [160], and PGAV [1]. Found naturally [133] and experimentally [225] infected with RVFV.

- ***Aedes* (*Neomelaniconion*) *fontenillei* (Le Goff, Boussès, & Brunhes, 2007)**

Le Goff, Boussès, and Brunhes, 2007 [143]

Endemic. Only adult stages (male and female) were described to date [143]. Sequence variations in ribosomal DNA ITS2 consistent with morphological observations, indicating that this species belongs to the Group Sylvaticum [130]. No specific differentiation at molecular level obtained between *Ae*. *fontenillei* and *Ae*. *sylvaticum*. In Madagascar, larval habitats may be small depressions in forest areas [143]. Adults collected from humans or using a hand-net in forest undergrowth. Only occurs in the Périnet forest, in the eastern biogeographic domain [143]. No medical and veterinary importance in Madagascar.

- ***Aedes* (*Neomelaniconion*) *nigropterum* (Le Goff, Boussès, & Brunhes, 2007)**

Le Goff, Boussès, & Brunhes, 2007 [143]

Endemic. Only the adult stages (male and female) were described to date [143]. Sequence variations in ribosomal DNA ITS2 consistent with morphological observations, indicating that this species belongs to the Group Circumluteolus [130]. No specific differentiation at molecular level obtained between this species and *Ae*. *belleci*. Only occurs in the Périnet forest, in the eastern biogeographic domain [143]. Adults were collected from humans or using a hand-net in forest undergrowth. No medical and veterinary importance in Madagascar.

- ***Aedes* (*Neomelaniconion*) *sylvaticum* (Le Goff, Boussès, & Brunhes, 2007)**

Le Goff, Boussès, & Brunhes, 2007 [143]

Endemic. Only the adult stages (male and female) were described [143]. Sequence variations in ribosomal DNA ITS2 consistent with morphological observations, indicating that this species belongs to the Group Sylvaticum [130]. No specific differentiation at molecular level obtained between this species and *Ae*. *fontenillei*. Probably present in the northern and eastern biogeographic domains [143]. Occurs in Sainte-Marie island, on the eastern edge of Madagascar (from Sambava region to Manakara region), and in medium mountainous areas (900 m asl). Collected using a hand-net in forest undergrowth. No medical and veterinary importance in Madagascar.

#### 2.3.8 Subgenus *Ochlerotatus* Lynch Arribálzaga, 1891

This subgenus includes 187 species in the world [241]. Only *Ae.* (*Och.*) *ambreensis* is present in Madagascar. The report of *Aedes dufouri* as present in Madagascar in Arim dataset [5] is doubtful as information on collection areas is not available. *De facto* this information was treated as an error. *Aedes dufouri* was described from Réunion Island and occurs on Europa island (French island in the Mozambique Channel) [99].

- ***Aedes* (*Ochlerotatus*) *ambreensis* Rodhain & Boutonnier, 1983**

Rodhain & Boutonnier, 1983 [193]

Endemic. Only the female was described [193]. Occurs in the Montagne d’Ambre, in the northern domain [85, 193]. Nocturnal, diurnal species and anthropophilic species, but also seems to feed on lemurs (*Eulemur fulvus*) [85]. Found naturally infected with unclassified virus (MMP 158 virus) in specimens collected at Montagne d’Ambre [85].

#### 2.3.9 Subgenus *Polyleptiomyia* Theobald, 1905

In Madagascar, this subgenus includes only a single species.

- ***Aedes* (*Polyleptiomyia*) *albocephalus* (Theobald, 1903)**

Grjebine, 1953 [103]

Eggs undescribed. In Madagascar, larval habitats are grassy bottom-land, watercourses, crab holes [103], grassy marshes, mangroves, and small collections of rainwater on salty soil connected to crab holes [182]. Occurs in all bioclimatic Malagasy domains (except the central domain) [85]. Anthropophilic [85], may be attracted to domestic ruminants (goat and cattle) (Luciano Tantely, unpublished observation). Not involved in disease transmission in Madagascar.

#### 2.3.10 Subgenus *Skusea* Theobald, 1903

The species of this subgenus only occur in the western Indian Ocean region. This subgenus includes four species, and three of them occur in Madagascar with one endemic species to the island. In this subgenus, *Aedes pembaensis*, absent from Madagascar, was ranked among the mosquito species found on the island [5, 244].

- ***Aedes* (*Skusea*) *cartroni* (Ventrillon, 1906)**

Ventrillon, 1906 [234]

Endemic to Madagascar and the Comoros archipelago. Egg, larval and pupal stages undescribed. Considered to be a synonym of *Aedes pembaensis* [80], absent from Madagascar. Presence in Madagascar validated by comparing male genitalia of the two species [25]. Larval habitats are probably brackish waters [182], including mangrove and swamps [85]. Occurs in the western biogeographic and southern domains [25]. Adult mosquitoes, probably belonging to this species, were collected in the northern and eastern domains [85]. For *Aedes cartroni*, human landing catches were productive close to brackish water [85]. Found naturally infected with MgV [85].

- ***Aedes* (*Skusea*) *lambrechti* van Someren, 1971**

Ravaonjanahary and Brunhes, 1977 [184]

Eggs undescribed. First described from the granitic Seychelles. Its larval habitats are small collections of rainwater on salty soil communicating with crab holes [182]. Occurs in Nosy Be and Nosy Komba, in Sambirano area [85], and in the northern biogeographic domain to Antalaha [182]. Not involved in disease transmission.

- ***Aedes* (*Skusea*) *moucheti* Ravaonjanahary & Brunhes, 1977**

Ravaonjanahary & Brunhes, 1977 [184]

Endemic. Only the adult male was described [184]. Larval habitats are crab holes filled with brackish water [184]. Occurs in Nosy Be [184] and in the western domain [15]. Not involved in disease transmission.

#### 2.3.11 Subgenus *Stegomyia* Theobald, 1901

This subgenus is represented by 126 species [241]. The Ethiopian region includes 59 species [125]. Only *Aedes albopictus* and *Ae*. *aegypti*, two invasive species, occur in Madagascar. *Ae.* (*Stg.*) *pia* (Le Goff & Robert), absent from Madagascar (this study, [5]), was ranked by WRBU among the mosquito species found on the island [244].

- ***Aedes* (*Stegomyia*) *aegypti* (Linnaeus, 1762)**

Bigot, 1859 [12]

In Madagascar, larval habitats are tree holes [103], peridomestic, tires, cans, metal drums, vehicle carcasses, small receptacles, and tree holes filled with rainwater and plant matter [182]. Occurs in all Malagasy biogeographic domains, with a high density in the western and southern domains where a large number of specimens were captured using human landing [85, 177]. In Madagascar, found naturally infected with BABV, MMP 158 virus, and WNV [85]. Known worldwide as vector of YFV [204], DENV [38], ZIKV [153], CHIKV [212], and at least 16 viruses [1]. Field vertical transmission of YFV [92] and DENV was already described for this species. Extrinsic development of ONNV was also described [231].

- ***Aedes* (*Stegomyia*) *albopictus* (Skuse, 1894)**

Ventrillon, 1905

In Madagascar, larval habitats are natural and artificial containers [small receptacles, tires, tree holes (coffee), cut bamboo, drums, cans] and leaf axils of *Pandanus* [182]. Occurs in all Malagasy biogeographic domains [85]. Currently expanding its geographic distribution in Madagascar, to the detriment of *Aedes aegypti* [177]. Found naturally infected with BABV in Madagascar [85]. Known worldwide as potential vector of SINBV [60], Cache Valley virus (CVV) [166], La Crosse virus (LACV) [94], Potosi virus (POTV) [6], CHIKV, DENV [55], and Banna virus (BAV) [148]. Involved in transmission of WNV in North America [8].

#### 2.3.12 Subgenus *Zavortinkius* Reinert, 1999

This subgenus includes 11 species [241]. Four species occur in Madagascar with three endemic species. Before creating the subgenus *Zavortinkius*, the Malagasy species were considered to belong to the subgenus *Finlaya*.

- ***Aedes* (*Zavortinkius*) *brygooi* Brunhes, 1971**

Brunhes, 1971 [22]

Endemic. Eggs undescribed. Larval habitats are tree holes full of plant organic matter [182]. Occurs essentially in warm regions characterized by a long dry season, and in Nosy Komba [90] and in all Malagasy biogeographic domains, with the exception of the eastern domain [85, 182]. Not involved in disease transmission.

- ***Aedes* (*Zavortinkius*) *interruptus* Reinert, 1999**

Reinert, 1999 [187]

Endemic. Only the adult stages (male and female) were described [187]. Larval habitats are water-filled trees [207]. Adult biology unknown. Occurs in the eastern [187] and central [210] biogeographic domains. Not involved in disease transmission.

- ***Aedes* (*Zavortinkius*) *monetus* Edwards, 1935**

Edwards, 1935 [79]

Eggs and pupal stages undescribed. First reported in Madagascar and was also collected on the islands of Comoros, Mayotte, and Moheli [24]. Larval habitats are tree holes filled with rainwater and plant organic matter [182]. Occurs in all Malagasy biogeographic domains (except the central domain) [85, 182]. Not involved in disease transmission.

- ***Aedes* (*Zavortinkius*) *phillipi* van Someren, 1949**

van Someren, 1949 [229]

Endemic. Eggs undescribed. Larval habitats are sectioned trunks of *Ravenala* sp. [22], tree holes [207], rarely leaf axils of *Pandanus* [182], bamboo ovitraps, and leaf axils of agave [85]. Essentially present in the warmer and humid eastern coast of Madagascar, also occurs in Nosy Komba, in the Sambirano area, western and northern [85] and the central [210] domains. Not involved in disease transmission.

### 2.4 Genus *Coquillettidia* Dyar, 1905 [71]

This genus includes 57 species in the world [117]. These species represent three subgenera: *Coquillettidia*, *Rhynchotaenia*, and *Austromansonia*. In Madagascar, only the subgenus *Coquillettidia* is present and it is represented by three species. Two of them are endemic to Madagascar. Larval and pupal stages of *Coquillettidia* species derive their oxygen by puncture of the aerenchyma of aquatic plants. The report of *Coquillettidia* (*Coquillettidia*) *aurites* (Theobald) as present in Madagascar in Arim dataset [5] is doubtful as information on collection areas is not available. *De facto* this information was treated as an error.

- ***Coquillettidia* (*Coquillettidia*) *grandidieri* (Blanchard, 1905)**

Ventrillon, 1904 [232]

Endemic. Eggs and pupal stages undescribed. Larval habitats are flushing holes containing clear water and floating aquatic plants [140]. Occurs in the western, eastern, and central biogeographic domains [85, 210]. Anthropophilic [85] and zoophilic species (feeds on domestic ruminants) [210]. RVFV was found in a mixed batch of mosquito species, which included *Cq. grandidieri*, collected in Périnet [85].

- ***Coquillettidia* (*Coquillettidia*) *metallica* (Theobald, 1901) [** **214** **]**

Doucet, 1951 [67]

Eggs undescribed. Larval habitats are unknown. In Madagascar, occurs in the western, eastern, and central domains and frequently caught in human landing catches [85]. In Madagascar, not involved in transmission of vector-borne disease. In Africa, involved in transmission of WNV [127], BABV, MIDV [1], and avian *Plasmodium* parasite [171].

- ***Coquillettidia* (*Coquillettidia*) *rochei* (Doucet, 1951) [** **67** **]**

Doucet, 1951 [67]

Endemic. Only adult males and females were described [67]. Larval habitats are unknown. Occurs at low altitude, in the western and eastern domains [67, 85, 140], with the exception of the forest corridor Anjozorobe-Angavo where this species was collected at altitudes below 1000 m asl [210]. Anthropophilic [85] but not involved in transmission of vector-borne diseases.

### 2.5 Genus *Culex* Linnaeus, 1758

The genus *Culex* includes 26 subgenera and 769 species in the world [119]. In total, 45 species from 6 subgenera were described in Madagascar. They include two *Culex salisburiensis* subspecies (*Culex salisburiensis salisburiensis* and *Culex salisburiensis coursi*). In Madagascar, like in many regions of the world, the systematics of *Culex* have to be revisited. The majority of these species belong to the subgenus *Culex*. Ten species are endemic to Madagascar. *Culex salisburiensis coursi* was described only from a single specimen.

#### 2.5.1 Subgenus *Culex* Linnaeus, 1758

This subgenus includes 198 species in the world. Twenty-seven species were recorded in Madagascar. Among them, *Culex scottii* Theobald and *Cx. vansomereni* Edwards were inventoried in this study and in the Arim dataset [5]. Three species are endemic to Madagascar and two other species occur on several Indian Ocean islands. The report of *Cx. sinaiticus* Kirkpatrick in the Arim dataset [5] and *Cx. thalassius* Theobald in WRBU [244] as present in Madagascar is doubtful as information on collection areas is not available. *De facto* this information was treated as an error.

- ***Culex* (*Culex*) *antennatus* (Becker, 1903)** [11]

Edwards, 1920 (as *Cx. laurenti*) [75]

This species belongs to Subgroup Decens in the Group Pipiens [115]. Larval stages are morphologically close to those of *Cx. decens* Theobald [124] and *Cx. quasiguiarti* Theobald [230]. Often confused with *Cx. univittatus* Theobald and *Cx. trifoliatus* Edwards [230], which is absent in Madagascar. In Madagascar, larval habitats are the hoof prints, canals, marshes, ditches, water reservoirs [63] and rice fields [67, 191]. Occurs in the Sambirano area (Nosy Be, Nosy Komba) [90], and all Malagasy biogeographic domains, except the northern domain [85, 181]. Zoophilic species and prefers to feed on cattle [210]. Frequently caught in the human landing catches in the central highlands [85] and may be attracted to poultry [210]. Involved in the transmission of *Wuchereria bancrofti* [20], RVFV [181, 210], WNV, and PERV [85]. BABV was isolated from a mixed batch of mosquito species, which included *Cx. antennatus*, collected in Périnet [85]. In Africa, zoophilic species (especially livestock) and occasionally feeds on humans [114]. Involved in transmission of NGAV [101], BABV, BAGV, WSLV, WNV [1], and *Setaria* sp. [16]. Sporozoites of avian *Plasmodium* parasite were reported in *Cx. antennatus* [199].

- ***Culex* (*Culex*) *argenteopunctatus* (Ventrillon, 1905)** [233]

Ventrillon, 1905 [233]

Endemic. Eggs undescribed. Larval habitats are wetlands [18], rice fields [67], grassy holes [30], puddles, and ponds [85]. Occurs in the central [85, 210] and eastern domains [67]. *Culex argenteopunctatus* exhibits strong positive phototropism using light traps, even within urban areas [85]. Not involved in the transmission of diseases.

- ***Culex* (*Culex*) *carleti* Brunhes & Ravaonjanahary, 1971** [29]

Brunhes and Ravaonjanahary, 1971 [29]

Endemic species to Madagascar and to the Comoros archipelago [29]. Eggs undescribed. Larval stages are morphologically close to *Cx. perfidiosus* Edwards and *Cx. mirificus* Edwards [29], which is absent in Madagascar. In Madagascar, larval habitats are cut bamboo [29]. Occurs in the Sambirano area (Nosy Be, Nosy Komba) [90] and in the eastern domain [85]. Captured during daytime catches in human landing catches [85]. Not involved in transmission of vector-borne diseases.

- ***Culex* (*Culex*) *comorensis* Brunhes, 1977** [24]

Brunhes, 1977 [24]

Endemic species to Madagascar and to the Comoros archipelago [24]. Eggs undescribed. Larval habitats are ovitraps, tree holes, puddles, ponds, and tire tracks [85]. Described in the Comoros archipelago, and collected by Brunhes in the Ankaratra Massif (1700 m asl) [24] and in the Andasibe-Mantadia forest (or forest Périnet), in the eastern domain [85]. Rare in Madagascar and not involved in the transmission of vector-borne diseases.

- ***Culex* (*Culex*) *decens* Theobald, 1901** [215]

Edwards, 1941 [80]

Eggs undescribed. Belongs to Decens Group, which is represented by *Cx. decens*, *Cx. quasiguiarti*, *Cx. guiarti* Blanchard, *Cx. scottii*, and *Cx. weschei* Edwards in Madagascar. The adult stages of these five species are morphologically close, and they are distinguishable by male morphology [85]. *Culex decens* and *Cx. invidiosus* Theobald are morphologically close, and few morphological differences were proposed in the larval [115] and adult stages [80]. In Madagascar, the breeding sites of *Cx. decens* include tree holes [103], ponds, rice fields, and swamps [67]. This species also grows in artificial ovitraps and bamboo [85]. This species is present in all Malagasy biogeographic domains [85]. *Culex decens* is abundant in the central domain [85, 210] and exhibited anthropophilic behavior in the village of Anjiro during the day [85]. This species exhibits positive phototropism using light traps [85] and may be attracted to livestock and poultry [210]. WNV and BABV were isolated from specimens caught in the Tsiroanomandidy area [85]. In Africa, this species was involved in the transmission of SINV, Usutu virus (USUV) [47], Moussa virus (MOUV) [175], BAGV, WNV, M’Poko virus (MPOV), Mossuril (MOSV), and Kamese virus (KAMV) [1].

- ***Culex* (*Culex*) *demeilloni* Doucet, 1950** [65]

Doucet, 1950 [65]

Endemic. Only larval stages were described [65]. The type was collected in a rice field in the Ambositra region [65]. This larval specimen has never been found in Madagascar since that time. Morphologically close to *Culex guiarti* by the presence of nine posterolateral combs of segment VIII and well visible teeth of the mentum [65].

- ***Culex* (*Culex*) *duttoni* Theobald, 1901** [215]

Edwards, 1941 [80]

Eggs undescribed. It is the only species of the Group Duttoni [115]. Its larval stages probably indistinguishable from those of *Culex watti* Edwards [124]. In Madagascar, larval habitats are puddles [103]. Occurs in the Sambirano area (Nosy Be) [90] and in a few localities of the central biogeographic domain [85]. Involved in the transmission of the Flavivirus in Africa (Yaoundé virus [YAOV], Ar 11266 B virus, UGSV) [1].

- ***Culex* (*Culex*) *grahamii* Theobald, 1910** [220]

Doucet, 1950 [65]

Eggs undescribed. Collected in Madagascar one time by Doucet in the Ambatolampy region [65]. The presence of this African species is questioned in Madagascar. This species can be confused with *Cx. striatipes* Edwards.

- ***Culex* (*Culex*) *guiarti* Blanchard, 1905**

Doucet, 1950 [65]

Eggs undescribed. In Madagascar, larval habitats are canals, ponds, rice fields, stagnant water ponds [67], and grassy holes [30]. Rare species and occurs only in the eastern domain [30, 67, 85]. Captured in human landing catches during night-time catches [85]. In Madagascar, not involved in transmission of vector-borne diseases. In Africa, involved in transmission of at least 12 viruses [1], for instance BAGV [45] and WNV [127], and Avian *Plasmodium* parasite [171].

- ***Culex* (*Culex*) *neavei* Theobald, 1906** [218]

Fontenille & Jupp, 1989 [88]

Eggs undescribed. Morphologically close to *Cx. univittatus*, also present in Madagascar. In Madagascar, larval habitats are still unknown. Presence reported only from the Tsiroanomandidy region [88]. Not involved in disease transmission. In Africa, may be attracted to domestic ruminants, poultry, and humans [10]. Involved in transmission of at least 16 viruses (Alphavirus, Flavivirus, Bunyaviruses, Orbivirus) [1] and avian *Plasmodium* parasite in Cameroon [171]. Seems to have a lower vectorial capacity than *Cx. univittatus* for the transmission of WNV [88].

- ***Culex* (*Culex*) *perfidiosus* Edwards, 1914**

Doucet, 1949 [64]

Egg and pupal stages undescribed. Larval stages might be confused with those of *Cx. carleti*, which were described in 1971 [29]. Larval habitats are in the rice fields, water holes, swamps, puddles, lakes, and flooded grasslands [63, 67]. In Madagascar, occurs in the eastern domain [63, 67].

- ***Culex* (*Culex*) *pipiens* Linnaeus, 1758**

Edwards, 1920 [75]

Eggs appear to be very similar in surface morphology to those of *Culex quinquefasciatus* Say [2]. In Madagascar, larval habitats are irrigation drains, canals, tires, and water tanks [207, 227]. In the central domain, occurs essentially in urban and suburban areas [227], also abundant in the Anorana rainforest, in the Anjozorobe district, in Antananarivo province [210]. A few specimens were recently collected in the western domain [15, 170]. Zoophilic species, prefers to feed on cattle and poultry. Not involved in disease transmission in Madagascar. In Africa, involved in transmission of RVFV [123], SINV [242], and avian *Plasmodium* parasite (presence of sporozoites) [199]. In North America, vector of WNV [8].

- ***Culex* (*Culex*) *quasiguiarti* Theobald, 1910** [220]

Edwards, 1941 [80]

Eggs undescribed. Morphologically close to *Culex decens* and *Cx. invidiosus*, and distinguishable by male genitalia morphology. In Madagascar, larval habitats are unknown. Occurs near Lake Alaotra (in the eastern domain) [80], in the Tsiroanomandidy region (central domain) [85], in the Ihosy and Ampandrandava regions (at the boundary between the southern and western domains) [80]. Not involved in the transmission of diseases.

- ***Culex* (*Culex*) *quinquefasciatus* Say, 1823**

Edwards, 1920 [80]

Eggs appear to be very similar in surface morphology to those of *Culex tritaeniorhynchus* Giles [205] and *Culex pipiens* [2]. Belongs to the subgroup pipiens of the Group Pipiens [115] and has about 40 synonyms. In Madagascar, larval habitats are sewage, tires, plastic containers, and metal drums (Tantely, unpublished data). Occurs in all geographic domains with a preference for urban environments [85]. Anthropophilic species [85] and may be attracted to domestic ruminants (Luciano Tantely, unpublished observation). Involved in the transmission of *Wuchereria bancrofti* [20], WNV, BABV, and endemic PERV [85]. In the world, has a variable host preference from high degree of mammalophily and anthropophily to a high degree of ornithophily [206]. Involved in transmission of RVFV [198], CHIKV, WNV, and MgV [1]. Involved in transmission of avian *Plasmodium* parasite (presence of sporozoites) in India and the United States of America [199].

- ***Culex* (*Culex*) *scottii* Theobald, 1912** [221]

Fontenille & Mathiot, 1984 [86]

Eggs, larval and pupal stages undescribed. Morphologically close to *Culex pipiens* Linnaeus, *Cx. musarum* Edwards, and *Cx. hancocki* Edward. *Culex musarum* and *Cx. hancocki* absent in Madagascar. Supposed to be endemic to the Seychelles [156], but occurs in Madagascar in the central (Mahasolo region) and in the eastern (Taolagnaro, Soanierana Ivongo, and Périnet) domains [85]. In Madagascar, anthropophilic and diurnal species [85] in the eastern and central domains [85]. WNV was isolated from specimens morphologically close to *Cx. scottii*, captured from an outdoor resting area near a cattle park in Mahazoarivo village (Mahasolo, Antananarivo province) [85].

- ***Culex* (*Culex*) *simpsoni* Theobald, 1905** [217]

Doucet, 1950 [65]

Eggs undescribed. Belongs to the subgroup Simpsoni of Group Pipiens [115]. In Madagascar, larval habitats are ponds [66], rock holes [103], fishponds, and tire tracks [85]. Occurs in the Sambirano area (in Nosy Be) [90] and in the eastern domain [66]. Rarely captured in human landing catches [85]. RVFV was found in a mixed batch of *Culex* mosquito species, including *Culex simpsoni*, collected in Périnet [85].

- ***Culex* (*Culex*) *sitiens* Wiedemann, 1828**

Edwards, 1941 [80]

Eggs undescribed. Belongs to the Group Sitiens [115]. In Madagascar, larval habitats are unknown, occurs in the Sambirano area (Nosy Be, Nosy Komba) [90], and in the western and central domains [85]. Not involved in disease transmission in Madagascar. In Africa, involved in transmission of MOSV in Mozambique [134], Murray Valley Encephalitis virus (MVEV), Japanese Encephalitis virus (JEV), Sepik virus (SEPV), and SINV in New Guinea [128]. In Malaysia, involved in the transmission of avian *Plasmodium* parasite (presence of sporozoites) [199].

- ***Culex* (*Culex*) *striatipes* Edwards, 1941** [80]

Brunhes & Ravaonjanahary, 1973 [30]

Egg and pupal stages undescribed. Larval stages are morphologically close to those of *Culex grahamii* Theobald [124]. In Madagascar, larval habitats are grassy swamps [30] and rice fields [191]. Occurs in the eastern and central domains, over 900 m asl [30]. Adult biology unknown and not involved in the transmission of diseases.

- ***Culex* (*Culex*) *theileri* Theobald, 1903** [216]

Doucet, 1951 [67]

Eggs undescribed. Belongs to Subgroup Theileri of Group Pipiens [115]. In Madagascar, presence reported with single female collected by Doucet in Vangaindrano area, on the eastern coast [67]. Presence is questioned in Madagascar.

- ***Culex* (*Culex*) *tritaeniorhynchus* Giles, 1901** [96]

Doucet, 1950 [65]

Eggs appear to be very similar in surface morphology to those of *Culex quinquefasciatus* [205]. Belongs to the Group Vishnui [115]. In Madagascar, larval habitats are in rice fields [67]. Occurs in all biogeographic domains of Madagascar. Frequently captured in human landing catches in the Morondava and Mahajanga regions [85]. Considered as a sylvatic vector of WNV [85]. MgV was isolated from this species [85]. In the world, major vector of JEV, SINV [242], NGAV and BABV [101], RVFV [129], Sagiyama virus (SAGV), Oya virus (OYAV), AKAV and Getah virus (GETV) [34], Yunnan orbivirus (YUOV) [7], and BANV [148]. In Japan, involved in transmission of avian *Plasmodium* parasite (presence of oocyst) [199].

- ***Culex* (*Culex*) *perfuscus* Edwards, 1914**

Grjebine, 1955 [5]

Eggs and pupal stages undescribed. No literature has reported its presence in Madagascar. However, this species occurs in Nosy Be, in the Sambirano area, as shown on the labels of specimens stored at the IRD of Montpellier [5]. Biology unknown. Not involved in transmission of diseases on the island. In Africa, involved in transmission of at least 19 viruses [1].

- ***Culex* (*Culex*) *trifilatus* Edwards, 1914**

Brunhes, 1971 [5]

Eggs, larval and pupal stages undescribed. No literature has reported its presence in Madagascar. However, this species occurs in Namakia, in the western domain, as shown on the labels of specimens stored at the IRD of Montpellier [5]. Biology unknown. Not involved in disease transmission.

- ***Culex* (*Culex*) *univittatus* Theobald, 1901** [215]

Ventrillon, 1905 [233]

Eggs undescribed. Belongs to the Group Pipiens [115]. Larval stages might be confused with those of *Cx. quasiguiarti* Theobald [230], *Cx. decens*, and *Cx. antennatus*. Adult stages morphologically close to *Cx. neavei*, also present in Madagascar. In Madagascar, larval habitats are rice fields and marshes [67]. Occurs in all Malagasy biogeographic domains (except the northern domain). Frequently found and abundant in the central highlands [85], exhibits positive phototropism [85]. In the central highlands, collected in net-traps baited with domestic ruminants and poultry [210]. In Madagascar, MgV was isolated from this species [85]. This species was found naturally infected with BABV in a mixed batch of mosquito species collected in Périnet [85]. In Africa, anthropophilic species and can also feed on cattle [114]. Involved in transmission of WNV [163], BABV [101], BAGV [165], RVFV [198], *Wuchereria bancrofti* [16], and avian *Plasmodium* parasite (presence of sporozoites) [199].

- ***Culex* (*Culex*) *ventrilloni* Edwards, 1920** [75]

Edwards, 1920 [75]

Endemic. Eggs, larval and pupal stages undescribed. Morphologically close to *Cx. simpsoni* and male genitalia of these two species are morphologically identical [80]. Presence only reported in Antananarivo city [85]. Further studies are needed to guarantee the status of this endemic species. Adult biology unknown. Not involved in disease transmission.

- ***Culex* (*Culex*) *watti* Edwards, 1920** [74]

Ravaonjanahary, 1979 [183]

Eggs undescribed. Larval stages morphologically close to those of *Culex duttoni* [124]. The larva of *Culex watti* is characterized by the presence of two subdorsal siphonal bristles which are absent in *Culex duttoni* [183]. In Madagascar, typical larval habitats are shady rock holes [183]. Occurs in the Sambirano area (Nosy Be, Nosy Komba) [90] and occasionally reported in the eastern domain [85]. Adult biology unknown. Not involved in disease transmission.

- ***Culex* (*Culex*) *vansomereni* Edwards, 1926** [77]

Clerc & Coulanges, 1979 [40]

Eggs undescribed. In Madagascar, larval habitats are unknown. Presence only reported in the Andasibe-Mantadia forest (or Périnet forest) in the eastern domain [40, 85]. Rare species. Adult biology unknown. BABV and RVFV were isolated from a mixed batch of mosquito species, including this species, collected in Périnet forest [85]. In Africa, ornithophilic species and competent vector of WNV under laboratory conditions [150]. Potential vector of RVFV [147].

- ***Culex* (*Culex*) *weschei* Edwards, 1935** [79]

Brunhes & Ravaonjanahary, 1973 [30]

Eggs and pupal stages undescribed. In Madagascar, larval habitats are grassy holes [30]. Occurs in the eastern and central domains [30, 85] and caught in human landing catches during daytime catches [85]. Not involved in disease transmission. In Africa, involved in transmission of WNV, MOSV, SINV [45], CHIKV, BABV, and WSLV. MgV was isolated from this species [1].

#### 2.5.2 Subgenus *Culiciomyia* Theobald, 1907 [219]

This subgenus includes 55 species in the world. Six species occur in Madagascar, two of them are endemic species to the island. The report of *Cx. semibrunneus* Edwards as present in Madagascar in the Arim dataset [5] is doubtful as information on collection areas is not available. *De facto* this information was treated as an error.

- ***Culex* (*Culiciomyia*) *cinerellus* Edwards, 1922** [76]

Grjebine, 1953 [103]

Eggs undescribed. Larval stages are morphologically close to those of *Cx. subaequalis* Edwards [124]. In Madagascar, larval habitats are tree holes [103] and many phytotelmata [27]. Occurs in the Sambirano area (Nosy Be, Nosy Komba) [90] and in the eastern domain. Caught in human landing catches during night-time catches [85]. Not involved in disease transmission.

- ***Culex* (*Culiciomyia*) *cinereus* Theobald, 1901**

Grjebine, 1953 [103]

Eggs undescribed. In Madagascar, larval habitats are tree holes [103]. Rare species and occurs in the western domain [103] and Sambirano area [85]. Adult biology unknown. In Africa, involved in transmission of at least 16 viruses [1].

- ***Culex* (*Culiciomyia*) *milloti* Doucet, 1949**

Doucet, 1949 [64]

Endemic. Only the larval stages were described [64]. Known only from a single specimen captured in the Tsimbazaza Park in Antananarivo [64]. Its larvae have similarities with those of *Culex nebulosus*. Larval habitats are water tables containing dissolved organic matter [64].

- ***Culex* (*Culiciomyia*) *nebulosus* Theobald, 1901**

Edwards, 1941

Eggs undescribed. In Madagascar, larval habitats are tree holes [103], bamboo trunks [85], and many phytotelmata [27]. Occurs in the Sambirano area (Nosy Be, Nosy Komba) [90], in the western, eastern, and central domains [85]. Captured in human landing catches [85], but not involved in disease transmission. In Africa, involved in transmission of Ntaya virus (NTAV) [17], BABV, MIDV, BAGV, YAOV, MPOV, and Tai virus (TAIV) [1].

- ***Culex* (*Culiciomyia*) *pandani* Brunhes, 1969** [21]

Brunhes, 1969 [21]

Endemic. Eggs undescribed. Larval habitats are cut trunks of *Ravenala* [29], tree holes, leaf axils of *Pandanus* [182] and agave [85], and many phytotelmata [27]. Rare species and occurs throughout the eastern cliffs of Madagascar [21, 85]. Captured in human landing catches during daytime catches [85]. Not involved in disease transmission.

- ***Culex* (*Culiciomyia*) *subaequalis* Edwards, 1941**

Brunhes, 1967 [5]

Eggs, pupal stages, and adult female undescribed. No literature has reported the presence of *Cx. subaequalis* in Madagascar. Occurs on the island, as shown on the labels of specimens stored at the IRD of Montpellier [5]. The locality type was not well specified. Biology unknown. Not involved in disease transmission.

#### 2.5.3 Subgenus *Kitzmilleria* Danilov, 1989

This subgenus includes only one species in the world: *Cx*. (*Kit*.) *moucheti*.

- ***Culex* (*Kitzmilleria*) *moucheti* Evans, 1923**

Coulanges et al., 1977 [48]

Eggs undescribed. Formerly classified in the subgenus *Culex*. In Madagascar, rare species and reported in the Andasibe-Mantadia forest (forested area near Périnet) in the eastern area [85]. Biology unknown. Not involved in disease transmission. In Africa, involved in transmission of NTAV [17].

#### 2.5.4 Subgenus *Oculeomyia* Theobald, 1907

This subgenus, rehabilitated by Tanaka in 2004, is represented by 19 species in the world. Five species are present in Madagascar and one species is endemic to the island. This study includes *Cx. aurantapex* Edwards that was not ranked among the mosquito species found on the island in WRBU [244]. The morphological diversity and the worldwide distribution of the species of subgenus *Oculeomyia* suggest that it is an old group [203]. Similar characters are observable in the larval stages for the majority of these species [124].

- ***Culex* (*Oculeomyia*) *annulioris* Theobald, 1901**

Clerc & Coulanges, 1980 [41]

Eggs undescribed. In Madagascar, larval habitats are unknown. Occurs in the eastern forest [41], central [210], western [15], and southern domains [170]. RVFV was isolated in a mixed batch of mosquito species, including *Cx. annulioris*, collected in Périnet forest [41]. In Africa, zoophilic species [114] and involved in transmission of SINV [237] and MIDV [1], and avian *Plasmodium* parasite (in Cameroon) [171].

- ***Culex* (*Oculeomyia*) *aurantapex* Edwards, 1914**

Brunhes, 1975

Eggs, pupal stages, and adult male undescribed. In Madagascar, larval habitats are unknown. Occurs only in the eastern area. Specimens collected at Périnet should be re-examined as all species close to Group Annulioris [85]. Captured in human landing catches during daytime catches [85], but not involved in disease transmission.

- ***Culex* (*Oculeomyia*) *bitaeniorhynchus* Giles, 1901**

Doucet, 1950 [65]

Eggs undescribed. In Madagascar, larval habitats are flooded grasslands [63], puddles [65], cattle hoof prints, swamps, rice fields, and ponds [67, 191]. Occurs in the western, eastern, central [85] and southern (in the Tsihombe region) biogeographic domains [65]. Captured in human landing catches [85], but not involved in disease transmission. In Africa, involved in transmission of SINV [196], SAGV, GETV [34], and RVFV [198]. In Japan, involved in transmission of avian *Plasmodium* parasite (with sporozoites) [199].

- ***Culex* (*Oculeomyia*) *giganteus* Ventrillon, 1906**

Ventrillon, 1906

Endemic. Eggs undescribed. Classified in the Bitaeniorhynchus Series of Group Lasioconops [78]. Currently, Harbach ranks this species in the subgenus *Oculeomyia* [119], contrary to what is indicated in Arim [5]. In Madagascar, larval habitats are grassy swamps, rice fields, and slow-flowing streams [18]. Occurs in the central domain [85, 210] and abundant on the eastern margins, in the medium altitude forest of Ranomafana (Fianarantsoa) and Périnet forest (or Andasibe-Mantadia forest) [85]. Recently observed in the western and southern domains [170]. May be attracted to domestic ruminants, poultry, and humans [210], but not involved in disease transmission.

- ***Culex* (*Oculeomyia*) *poicilipes* (Theobald, 1903)**

Ventrillon, 1905 [233]

Egg and pupal stages undescribed. Morphologically atypical species, and classified in the subgenus *Oculeomyia* [244], contrary to what is indicated in Arim [5]. In Madagascar, larval habitats are ponds and rice fields [67, 103]. Occurs particularly in the central and eastern domain and collected in abundance around lake Soamalipo, in the Antsalova region of the western domain [15]. May be attracted to humans and poultry bait [85] (Luciano Tantely, unpublished observation). Not involved in disease transmission. In Africa, zoophilic species and feeds on livestock, birds, and humans [114]. Involved in transmission of NGAV [101], WNV, BAGV [222], RVFV, SANV [58], *Setaria* sp. [16], and avian *Plasmodium* parasite (in Cameroon) [171].

#### 2.5.5 Subgenus *Eumelanomyia* Theobald, 1909

This subgenus includes 77 species in the world [119]. Among which eight species are present in Madagascar with two endemic species to the island, and *Cx. sunyaniensis* Edwards that was not ranked by WRBU among the mosquito species of the island [244].

- ***Culex* (*Eumelanomyia*) *brenguesi* Brunhes & Ravaonjanahary, 1973** [30]

Brunhes & Ravaonjanahary, 1973 [30]

Endemic. Eggs undescribed. Belongs to Group Rubinotus-rima [30]. Larval stages are morphologically close to those of *Cx. sunyaniensis*. Larval habitats are grassy holes and swamps [30]. Rare species and occurs in the eastern domain [30]. Adult biology unknown. Not involved in disease transmission.

- ***Culex* (*Eumelanomyia*) *chauveti* Brunhes & Rambelo, 1968**

Brunhes & Rambelo, 1968 [28]

Endemic to Madagascar and to the Comoros archipelago (Mohéli) [28]. Eggs undescribed. Larval habitats are temporary pools and small grassy pools under forest cover [28, 143]. Occurs on the eastern slopes of the central highlands and in the eastern domain [28, 85]. Captured in human landing catches during daytime catches [85]. Not involved in disease transmission.

- ***Culex* (*Eumelanomyia*) *horridus* Edwards, 1922**

Grjebine, 1953 [103]

Eggs undescribed. Belongs to the Group Protomelanoconion [202]. Includes two subspecies: the only subspecies *Cx. horridus* is present in Madagascar. Larval habitats are tree holes [103]. Occurs in the western [103] and central domains [85]. Adult biology unknown. Not involved in disease transmission.

- ***Culex* (*Eumelanomyia*) *insignis* (Carter, 1911)**

Grjebine, 1955 [5]

Eggs undescribed. No literature has reported its presence in Madagascar. However, this species occurs in Nosy Be, in the Sambirano area, as shown on the labels of specimens stored at the IRD of Montpellier [5]. Biology unknown. Not involved in disease transmission.

- ***Culex* (*Eumelanomyia*) *kingianus* Edwards, 1922**

Doucet, 1950 [65]

Eggs undescribed. In Madagascar, larval habitats are tree holes [85], and phytotelmata [27], fresh water marshes (in Mahajanga city) [112]. Occurs in the western [103] and eastern domains [85]. Adult biology unknown. Not involved in disease transmission.

- ***Culex* (*Eumelanomyia*) *rubinotus* Theobald, 1906**

Fontenille & Mathiot, 1984 [89]

Eggs undescribed. In Madagascar, larval habitats are tree holes [85] and many phytotelmata [27]. Occurs in the Sambirano area (Nosy Be) [90] and in the eastern domain [85]. Not involved in disease transmission. In Africa, involved in transmission of RVFV [147], UGSV, Germiston virus (GERV) [1], Banzi (BANV), and Witwatersrand (WITV) virus [162].

- ***Culex* (*Eumelanomyia*) *sunyaniensis* Edwards, 1941**

Doucet, 1950 [65]

Eggs undescribed. In Madagascar, larval habitats are slow streams of Périnet forest and in leaf axils of *Pandanus* located in the Vangaindrano region of the eastern domain [66]. Its presence must be confirmed by observing male genitalia morphology. Adult biology unknown. Rare species, not involved in disease transmission.

- ***Culex* (*Eumelanomyia*) *wigglesworthi* Edwards, 1941**

Grjebine, 1952 [5]

Egg and pupal stages undescribed. No literature has reported its presence in Madagascar. However, this species occurs in Manakara, eastern domain, as shown on the labels of specimens stored at the IRD of Montpellier [5]. Biology unknown. Not involved in disease transmission.

#### 2.5.6 Subgenus *Maillotia* Theobald, 1907

This subgenus includes nine species in the world [119]. Two species of this subgenus are reported in Madagascar. The report of *Cx. avianus* de Meillon as present in Madagascar in the WRBU dataset [244] is doubtful as information on collection areas is not available. *De facto* this information was treated as an error.

- ***Culex* (*Maillotia*) *salisburiensis* Theobald, 1901**

Doucet, 1949 [63]

In Madagascar, presence reported of two *Culex salisburiensis* subspecies (*Culex salisburiensis salisburiensis* and *Culex salisburiensis coursi*). The subspecies *Culex salisburiensis coursi*, endemic [63], described only from a single specimen and known only at larval stages collected from rice fields in the eastern domain. Sympatric with *Culex salisburiensis salisburiensis* in the Lake Alaotra region on the eastern slope of the central highlands [63]. For *Culex salisburiensis salisburiensis*, pupal stage and eggs undescribed. Adult biology unknown. Not involved in the transmission of diseases.

- ***Culex* (*Maillotia*) *seyrigi* Edwards, 1941** [80]

Edwards, 1941 [80]

Endemic. Eggs undescribed. In Madagascar, larval habitats are swamps [18], rock holes and grassy ditches [19]. Occurs in the central domain [19]. Adult biology unknown. Not involved in disease transmission.

### 2.6 Genus *Eretmapodites* Theobald, 1901

The genus *Eretmapodites* includes 48 species that occur only in Afrotropical region [117, 119]. Four species were reported in Madagascar.

- ***Eretmapodites oedipodeios* Graham, 1909**

Doucet, 1950 [65]

Eggs, larval and pupal stages undescribed. Presence reported in Madagascar by Doucet, only in 1950 from specimens caught by Paulian in Taolagnaro [65]. In Africa, involved in transmission of Eret 147 virus in Cameroon [1].

- ***Eretmapodites plioleucus* Edwards, 1941**

Doucet, 1950 [65]

Eggs, larval and pupal stages undescribed. Includes two subspecies: *Er. plioleucus brevis* and *Er. plioleucus plioleucus*. Morphologically close to *Er. leucopous* Graham which is absent from Madagascar. In Madagascar, presence reported only in 1950 by Doucet, from specimens caught by Paulian on Europa island and in the Lokobe region of the Sambirano area [65]. Its presence on the Indian Ocean islands is questioned. Not involved in disease transmission.

- ***Eretmapodites quinquevittatus* Theobald, 1901 [** **214** **]**

Ventrillon, 1905 [233]

In Madagascar, larval habitats are stagnant water [65] and many phytotelmata [27]. Occurs in all Malagasy biogeographic domains [85]. Rare species in the central domain [85]. Anthropophilic and diurnal species under forest area [85]. MgV was isolated from *Er. quinquevittatus* [90]. In Africa, involved in transmission of RVFV [174] and viruses belonging to the genera Flavivirus and Bunyaviruses [1].

- ***Eretmapodites subsimplicipes* Edwards, 1914**

Doucet, 1951 [66]

Eggs undescribed. In Madagascar, presence reported only in 1951 by Doucet in Périnet forest in the eastern domain [66]. Adult and larval biology unknown. Rare species and not involved in disease transmission. In Comoros archipelago, anthropophilic and nocturnal species [24]. In Kenya, involved in transmission of Okola virus (OKOV) [1].

### 2.7 Genus *Ficalbia* Theobald, 1903

The genus *Ficalbia* belongs to the tribe Ficalbiini with the genus *Mimomyia*. The genus *Ficalbia* is represented by only eight species in the world [119]. Four species occur in the Afrotropical region. In Madagascar, this genus is represented by two species. Among them, *Fi. circumtestacea* was not reported as present on the island by WRBU [244]. Some specimens collected in other bioclimatic domains by Fontenille [85] could not be identified with confidence. This observation suggests that this genus is probably insufficiently studied in Madagascar.

- ***Ficalbia uniformis* (Theobald, 1904)**

Doucet, 1949 [63]

Larval habitats are flooded meadows, marshes, canals, and deep clear water containing abundant aquatic vegetation [63]. Occurs in the eastern and central domains [63]. Adult biology unknown. Not involved in disease transmission.

- ***Ficalbia circumtestacea* (Theobald, 1908)**

Grjebine, 1986 [110]

Eggs undescribed. In Madagascar, reported at the larval stage, in the eastern domain, in the Andasibe-Mantadia and Manakara regions [110] and adult stage in Antsalova district of the western domain [15]. Adult biology unknown. Not involved in disease transmission.

### 2.8 Genus *Hodgesia* Theobald, 1903

The *Hodgesia* is represented by 11 species in the world [119]. Four species occur in Afrotropical region, mainly in central Africa. Adult females of African specimens are indistinguishable [158]. In Madagascar, presence was reported by Fontenille [85] who captured seven adults in human landing catches during daytime catches in April 1984 in the Mandena forest (Taolagnaro) [85]. The Malagasy specimens are still unidentified to date. This is probably the reason why Arim and WRBU did not rank this genus among the mosquito genera found in Madagascar [5, 244]. Two females are currently stored in the laboratory of vector taxonomy of IRD Montpellier. Two other female specimens were caught by Didier Fontenille, in human landing catches, in May 1983, in the Antetezana forest, along the eastern coast between Toamasina and Foulpointe (Gilbert Le Goff, unpublished observation). Capture efforts for larval stages in swamp areas of the eastern coast could facilitate specimen collection and species identification. *Hodgesia* is poorly known, and rarely feeds on humans and is not known to be involved in medical or veterinary pathogen transmission.

### 2.9 Genus *Lutzia* Theobald, 1903

The genus *Lutzia* was formerly classified in the genus *Culex* and it was subdivided into three subgenera represented by eight species in the world [119]. Only one species belonging to the subgenus *Metalutzia* is present in the Afrotropical region and Madagascar.

#### 2.9.1 Subgenus *Metalutzia* Tanaka, 2003

This subgenus includes five species; one species occurs in Madagascar: *Lutzia tigripes.*

- ***Lutzia* (*Metalutzia*) *tigripes* de Grandpre & de Charmoy, 1901**

Edwards, 1920 [75]

Eggs undescribed. Larval stages are predators of mosquito-associated species and usually found in association with other species in many larval habitats. In Madagascar, larval habitats are canoes, marshes, canals [67], swamps [103], tires, puddles, flooded lowlands [207], and rice fields [191]. Occurs in the Sambirano area (Nosy Be, Nosy Komba) [90], in the western, eastern, and central domains [67, 85]. Not involved in disease transmission. In Africa, involved in transmission of NTAV [17], WNV [200], and many other viruses in the Central African Republic (SINV, BABV, Bobia virus [BIAV], MOSV, and KAMV) [1].

### 2.10 Genus *Mansonia* Blanchard, 1901

This genus is subdivided into two subgenera and includes 25 species in the world [119]. In Madagascar, only the subgenus *Mansonoides* occurs and it is represented by two species. Among them, *Ma. africana* was not reported to be present on the island [244]. Larvae grow in permanent waters containing aquatic plants and derive their oxygen by taking air from the aerenchyma of aquatic plants.

#### 2.10.1 Subgenus *Mansonoides* Theobald, 1907

- ***Mansonia* (*Mansonoides*) *africana* (Theobald, 1901)**

Grjebine, 1953 [103]

Eggs undescribed. Represented by two subspecies: the subspecies *Ma*. *africana nigerrima* confined to central Africa and *Ma*. *africana africana* present throughout the Afrotropical region and in Madagascar [120]. In Madagascar, occurs in the western domain [85, 103], anthropophilic [85]. In Africa, anthropophilic species [50]. Involved in transmission of SPOV [160], MIDV, PGAV, RVFV [45], RVFV [93], at least 13 arboviruses (Alphavirus, Flavivirus, Bunyaviruses, Phlebovirus) [1, 224] and *Brugia patei* [16].

- ***Mansonia* (*Mansonoides*) *uniformis* (Theobald, 1901)**

Edwards, 1920 [75]

In Madagascar, larval habitats are ponds and rice fields [67]. Occurs in all Malagasy biogeographic domains (except the northern domain) [85]. Abundant, anthropophilic [85], zoophilic, nocturnal, and crepuscular species (Luciano Tantely, unpublished observation). Involved in transmission of RVFV, BABV, PERV [85], WNV [152], *Wuchereria bancrofti* [111], *Setaria* sp. and *Dirofilaria* spp. [23]. In Africa, zoophilic species in some areas and anthropophilic species in others [114]. Occasionally feeds on birds and bats [114]. In the world, involved in transmission of MIDV, Yata virus (YATV) [45], ZIKV, CHIKV [47], WNV [127], ONNV [151], RVFV [198], at least 16 arboviruses (Alphavirus, Flavivirus, Bunyaviruses, Orbivirus, Rhabdovirus, Phlebovirus) [1], and avian *Plasmodium* parasite (in Cameroon) [171].

### 2.11 Genus *Mimomyia* Theobald, 1903

The genera *Mimomyia* and *Ficalbia* belong to the Ficalbiini tribe. The genus *Mimomyia* includes 45 species subdivided into three subspecies: *Etorleptiomyia* (7 species), *Ingramia* (21 species), and *Mimomyia* (17 species) [119]. In Madagascar, 22 species were reported, and 17 of them are endemic. The phylogenetic relationship between the genus *Mimomyia* and other Culicidae genus remains uncertain, and the morphological data suggest affinity with the genera *Ficalbia* and *Hodgesia* [121]. The biology of genus *Mimomyia* remains poorly known. The species of this genus have no medical or veterinary importance in Madagascar, although some species were found naturally infected with arboviruses, particularly in Senegal [1].

#### 2.11.1 Subgenus *Etorleptiomyia* Theobald, 1904

The subgenus *Etorleptiomyia* includes seven species, occurring mainly in the Ethiopian, eastern, and Australian regions. Two species were found in Madagascar, one being endemic to the island. The species of the subgenus *Ertoleptiomyia* breed in a wide variety of terrestrial water accumulations (marshes, ponds).

- ***Mimomyia* (*Etorleptiomyia*) *martinei* (Doucet, 1951)**

Doucet, 1951 [66]

Endemic. Only the adult female was described [66]. Its existence and its membership to one subgenus were repeatedly questioned [157]. Without being able to provide indisputable evidence, some authors suggest that the description of the female stage could correspond to that of *Mi.* (*Ingramia*) *spinosa* [110, 157]. If this were the case, these two species would be synonymous and retain the name *Mimomyia martinei* [110]. Pending further information, this species must be regarded as valid and inventoried in the Malagasy subgenus *Ertoleptiomyia* [119]. This endemic species was collected only once and is known only from the type locality (Périnet area) [66], where two adults were captured from an outdoor resting area, in hollow bamboo. This species was not found since that time.

- ***Mimomyia* (*Etorleptiomyia*) *mediolineata* (Theobald, 1904)**

Rodhain 1979 cited by [85]

Eggs undescribed. In Madagascar, larval habitats are coastal marshes, containing herbaceous plants (Cyperaceae, ferns), tannins, and plant organic matter [110]. Occurs in the Manakara and Taolagnaro regions (eastern domain), and the Mahajanga region (western domain) [85]. Captured in human landing catches [85]. In Africa, feeds mainly on amphibians and occasionally on humans [13].

#### 2.11.2 Subgenus *Ingramia* Edwards, 1912

In total, among the 21 species described in subgenus *Ingramia*, 16 species occur only in Madagascar.

- ***Mimomyia* (*Ingramia*) *aurata* (Doucet, 1951)**

Doucet, 1951 [66]

Endemic. Eggs undescribed. Belongs to a complex of six species (*Mi. aurata*, *Mi. bernardi*, *Mi. beytouti*, *Mi. collessi*, *Mi. marksae*, *Mi. mattinglyi*) which are practically indistinguishable in the adult stage. Exhibits differences, sometimes marked, in the larval and pupal stages. Morphologically close to *Mi. bernardi* and *Mi. beytouti*. Larval habitats are leaf axils of *Ravenala* fronds [66, 85, 110] and leaf axils of *Pandanus* [110]. Occurs in the central and eastern domains [66, 85, 110]. Diurnal species and captured in human landing catches [85]. Not involved in disease transmission.

- ***Mimomyia* (*Ingramia*) *bernardi* (Doucet, 1950)**

Doucet, 1950 [65]

Endemic. Eggs undescribed. Belongs to the complex of six species cited above. All developmental stages are morphologically close to those of *Mi. aurata*. Larval habitats are in axils of *Ravenala* and *Pandanus* fronds in forested area [65]. Occurs in a large part of the eastern domain [65]. Biology unknown. Not involved in the transmission of disease.

- ***Mimomyia* (*Ingramia*) *beytouti* (Doucet, 1951)**

Doucet, 1951 [67]

Endemic. Eggs undescribed. Belongs to the complex of six species cited above. Morphologically close to *Mi. collessi* in the larval stage. Shows significant differences in the pupal stage. Larval habitats are leaf axils of *Ravenala* [67, 110]. Occurs in eastern domain [110]. Biology unknown. Not involved in disease transmission.

- ***Mimomyia* (*Ingramia*) *brygooi* Grjebine, 1986**

Grjebine, 1986 [110]

Endemic. Adult female and eggs undescribed. Because of the similarity between the larval stages within the subgenus *Ingramia*, larval capture data may refer to one of the following species: *Mi. brygooi*, *Mi. levicastilloi*, and *Mi. longicornis*, or to *Mi. ramalai*. The only adult stage known is the two males used in the original description, they were obtained from larval rearing. Larval habitats are leaf axils of *Pandanus* and *Ravenala* [85]. Occurs in the eastern domain [110]. Adult biology unknown. Not involved in disease transmission.

- ***Mimomyia* (*Ingramia*) *collessi* Grjebine, 1986**

Grjebine, 1986 [110]

Endemic. Eggs undescribed. Belongs to the complex of six species cited above. Morphologically close to *Mi. beytouti*. Occurs mainly in the eastern domain [110]. Adult biology unknown. Not involved in disease transmission.

- ***Mimomyia* (*Ingramia*) *jeansottei* (Doucet, 1950)**

Doucet, 1950 [65]

Endemic. Eggs undescribed. Larval habitats are leaf axils of *Ravenala*, and *Nepenthes madagascariensis* pitchers of the coastal peatlands. Only occurs on the eastern coast of Madagascar [27, 109, 110]. Adult biology unknown. Not involved in disease transmission.

- ***Mimomyia* (*Ingramia*) *levicastilloi* Grjebine, 1986**

Grjebine, 1986 [110]

Endemic. Adult stage and eggs undescribed. Larval habitats are leaf axils of *Pandanus*. Occurs along the coastal dunes of the eastern domain [110].

- ***Mimomyia* (*Ingramia*) *longicornis* Grjebine, 1986**

Grjebine, 1986 [110]

Endemic. Eggs undescribed. Larval habitats are leaf axils of large *Pandanus*, in the forested zone. Adult biology unknown because adult stages were known only from larval rearing [110]. Collected only once and known only from the type locality (Ambodirina Forest), in the eastern domain, at the boundary between the eastern and central highlands. Not involved in disease transmission.

- ***Mimomyia* (*Ingramia*) *marksae* Grjebine, 1986**

Grjebine, 1986 [110]

Endemic. Eggs undescribed. Belongs to a complex of species morphologically closes, including *Mi. beytouti*, *Mi. collessi*, and *Mi. marksae*. Larval habitats are leaf axils of *Ravenala*, in the eastern domain. Adult biology unknown because adults were known only from larval rearing [110]. Not involved in disease transmission.

- ***Mimomyia* (*Ingramia*) *mattinglyi* Grjebine, 1986**

Grjebine, 1986 [110]

Endemic. Adult female and eggs undescribed. The only known male was obtained from larval holotype rearing. Larval habitats are leaf axils of *Ravenala*. Only occurs in the Andasibe forest of the eastern domain [110]. Adult biology unknown. Not involved in disease transmission.

- ***Mimomyia* (*Ingramia*) *milloti* Grjebine, 1986**

Grjebine, 1986 [110]

Endemic. Eggs undescribed. Belongs to a complex of species with *Mimomyia roubaudi* in Madagascar and *Mi. grjebinei* in the Comoros archipelago. Larval habitats are leaf axils of Arum (*Colocasia* sp. and *Typhonodorum*) and *Pandanu*s. Collected in the central domain [110]. Adult biology unknown. No medical and veterinary importance.

- ***Mimomyia* (*Ingramia*) *ramalai* Grjebine, 1986**

Grjebine, 1986 [110]

Endemic. Adult male and egg undescribed. The two females, used in the description, were obtained from larval rearing. Larval habitats are leaf axils of *Pandanus*. Collected only once and known only from the type locality (Mandraka forest). Morphologically close to *Mimomyia brygooi*. Adult biology unknown. Not involved in disease transmission.

- ***Mimomyia* (*Ingramia*) *roubaudi* (Doucet, 1950)**

Doucet, 1950 [65]

Endemic. Eggs undescribed. Belongs to a complex of species with *Mimomyia milloti* which occurs in Madagascar and *Mi. grjebinei* in the Comoros archipelago. Morphological variations observed from specimens collected in the Vohipeno region, allowing us to assume the presence of a complex of species. Larval habitats are mainly the leaf axils of *Typhonodorum* sp. and exceptionally the axils of fronds of *Ravenala*. Occurs in the eastern domain and locally on the west coast of Madagascar (Nosy Be, Morondava, and Mahajanga regions) [103]. Adult biology unknown. Not involved in disease transmission.

- ***Mimomyia* (*Ingramia*) *spinosa* (Doucet, 1951)**

Doucet, 1951 [66]

Endemic. Eggs undescribed. Redescription of specimens collected in Analamazaotra forest near Périnet forest, allowed Grjebine [110] to suggest that this species is a synonym of *Mimomyia martinei*, without providing any evidence [110]. Larval habitats are mainly axils of fronds of *Ravenala* and bamboo [66]. Occurs in the eastern domain [66, 110]. Adult biology unknown. Not involved in disease transmission.

- ***Mimomyia* (*Ingramia*) *stellata* Grjebine, 1986**

Grjebine, 1986 [110]

Endemic. Eggs undescribed. Larval habitats are axils of fronds of *Ravenala* and bamboo. Only occurs in the Moramanga region (Périnet, Mandraka, and Lakato) in forested areas of the eastern domain. Adults are known only from larval rearing [110]. Not involved in disease transmission.

- ***Mimomyia* (*Ingramia*) *vansomerenae* Grjebine, 1986**

Grjebine, 1986 [110]

Endemic. Only the larval stages were described [110]. Known only from the type locality (Lokobe Reserve, Nosy Be). Larval habitats are axils of fronds of *Ravenala* [110].

#### 2.11.3 Subgenus *Mimomyia* Theobald, 1903

The subgenus *Mimomyia* includes 21 species, widely distributed throughout the Ethiopian and Oriental regions and extends to northern Australia and the South Pacific. The four Malagasy species have a wide distribution on the African mainland. The report of *Mi. lacustris* Edwards and *Mi. pallida* Edwards as present in Madagascar in the Arim dataset [5] is doubtful as information on collection areas is not available. *De facto* this information was treated as an error. The species of the subgenus *Mimomyia* develop in a wide variety of terrestrial water accumulations (ponds, marshes, ponds, and riverbanks).

- ***Mimomyia* (*Mimomyia*) *hispida* (Theobald, 1910)**

Doucet, 1951 [66]

Eggs undescribed. In Madagascar, larval habitats are ponds and marshes containing abundant aquatic vegetation [110], muddy swamp water and rice fields with low vegetation [67]. Occurs in the central and eastern domains [65, 67, 85, 110]. Adult biology unknown. In Africa, caught in human biting catches in West Africa [155] and feeds mainly on amphibians and cattle in Kenya [13]. Involved in transmission of BABV, BAGV, and WNV [222].

- ***Mimomyia* (*Mimomyia*) *mimomyiaformis* (Newstead, 1907)**

Doucet, 1951

Eggs undescribed. In Madagascar, larval habitats are stagnant or slow moving water, with aquatic vegetation (swamps, irrigation canals, and rivers) within forested areas [66]. Occurs in the eastern domain, western coastal plains, in the Mahajanga region [110]. Adult biology unknown. In Africa, captured in human biting catches [155]. Not involved in disease transmission.

- ***Mimomyia* (*Mimomyia*) *plumosa* (Theobald, 1901)**

Doucet, 1951 [67]

Eggs undescribed. In Madagascar, larval habitats are forest ponds with vegetation [110]. Occurs in the eastern domain (Périnet and Vangaindrano) and the Sambirano area. Adult biology unknown. Not involved in disease transmission. Adult biology unknown. In Africa, involved in transmission of *Bunyaviruses* of the Bwamba Group, a non-pathogenic virus to humans [1].

- ***Mimomyia* (*Mimomyia*) *splendens* Theobald, 1903**

Grjebine, 1956

Eggs undescribed. In Madagascar, larval habitats are terrestrial water accumulations, invariably associated with aquatic plants, indispensable for breathing larvae. Occurs throughout the eastern domain, from the eastern margin of the central highlands (Moramanga Périnet) to the coastal lagoons of the south-eastern domain (Manakara and Vangaindrano regions). Also reported locally in the western domain (Mahajanga region). In Africa, feeds mainly on amphibians and occasionally on humans [13]. Involved in transmission of WNV, BABV, and BAGV [1].

### 2.12 Genus *Orthopodomyia* Theobald, 1904

This genus is represented by 36 species in the world [119]. Eight species occur in Madagascar. The Malagasy species belong to Group Vernoni and are all endemic to Madagascar. They are not involved in disease transmission.

- ***Orthopodomyia ambremontis* Brunhes & Hervy, 1995 [** **27** **]**

Brunhes & Hervy, 1995 [27]

Endemic. Eggs and adult female undescribed. Larval habitats are tree holes. Occurs in the Montagne d’Ambre, at altitudes above about 1200 m asl, in the northern domain [27]. Adult biology unknown.

- ***Orthopodomyia ankaratrensis* Brunhes & Hervy, 1995 [** **27** **]**

Brunhes & Hervy, 1995 [27]

Endemic. Eggs, pupal and adult stages undescribed. Larval habitats are tree holes. Collected only once and known only from the type locality (Manjakatompo, Ankaratra massif), at altitudes above about 1800 m, in the central domain. Adult biology unknown [27].

- ***Orthopodomyia fontenillei* Brunhes & Hervy, 1995 [** **27** **]**

Brunhes & Hervy, 1995 [27]

Endemic. Eggs undescribed. Larval habitats are tree holes. Occurs in forested areas, at an altitude greater than 80 m asl of the eastern [27] and central domains [207]. Adult biology and larval habitats unknown.

- ***Orthopodomyia milloti* Doucet, 1951 [** **66** **]**

Doucet, 1951 [66]

Endemic. Eggs undescribed. Larval habitats are tree holes, leaf axils of *Pandanus* and *Ravenala* and bamboo [85, 182, 207], and ovitraps [85]. Occurs in the eastern and central domains and seems to be frequent at lower altitudes (below 800–900 m): from the sea to the eastern margins of the central highlands [27, 85]. Adult biology unknown.

- ***Orthopodomyia rajaonariveloi* Brunhes & Hervy, 1995 [** **27** **]**

Brunhes & Hervy, 1995 [27]

Endemic. Only known from the holotype female [27]. Biology unknown. Occurs only in the Fenoarivo Atsinanana region of the eastern domain [27].

- ***Orthopodomyia ravaonjanaharyi* Brunhes & Hervy, 1995 [** **27** **]**

Brunhes & Hervy, 1995 [27]

Endemic. Eggs undescribed. Larval habitats are tree holes (*Albizzia*, mango tree). Only occurs in the Sambava and Antalaha regions, on the north-eastern coast of Madagascar. Adult biology unknown.

- ***Orthopodomyia rodhaini* Brunhes & Hervy, 1995 [** **27** **]**

Brunhes & Hervy, 1995 [27]

Endemic. Egg and adult stages undescribed. Larval habitats are tree holes and cut bamboo. Only occurs in Antongil Bay, in the eastern domain [27].

- ***Orthopodomyia vernoni* van Someren, 1949 [** **229** **]**

van Someren, 1949 [229]

Endemic. Eggs undescribed. Larval habitats are many phytotelmata but sometimes artificial containers (tin cans and metal cans) [27, 85]. Occurs in the western and southern domains of Madagascar, and can reach areas bordering other bioclimatic domains, at lower altitude (below 1000 m).

### 2.13 Genus *Toxorhynchites* Theobald, 1901

This genus includes four subgenera represented by 89 species in the world [119]. Six species occur in Madagascar. This study did not include *Tx. brevipalpis* that is reported to be present on the island by WRBU [244]. The Malagasy species belong only to the subgenus *Afrorhynchus* which dominates on the African mainland, and within the Group Pauliani which is endemic to Madagascar [190]. The external morphology of this group is homogeneous. Only male genitalia morphology allows differentiation of these species. The adult stages of *Toxorhynchites* are phytophagous and are not involved in transmission of pathogens. The larval stages are predators feeding on larval stages of other mosquito species. Its host preference allows us to consider *Toxorhynchites* mosquitoes as a biological control agent of vector mosquitoes [190]. In Madagascar, *Toxorhynchites* larva develops generally in many phytotelmata: *Typhonodorum*, *Ravenala*, *Pandanus*, *Nepenthes madagascarensis*, *Colocasia*, bamboo, and fruit shells [111]. Most Malagasy *Toxorhynchites* species occur in the eastern biogeographic domain [67, 190]. Larval stages of this genus were collected by Rodhain et al. [195] in the Mahajanga area, of the western domain, but remained unidentified. The adult biology of *Toxorhynchites* of Madagascar is unknown and the Malagasy species has no medical or veterinary importance.

- ***Toxorhynchites* (*Afrorhynchus*) *brunhesi* Ribeiro, 2004 [** **190** **]**

Ribeiro, 2004 [190]

Endemic. Eggs undescribed. The holotype male was collected in the Moramanga district located in the eastern domain, on the eastern margin of the central highlands.

- ***Toxorhynchites* (*Afrorhynchus*) *fontenillei* Ribeiro, 2004 [** **190** **]**

Ribeiro, 2004 [190]

Endemic. Eggs, larval and pupal stages and adult female undescribed. The holotype male was obtained from larval rearing after collection in *Ravenala*, in the rainforest park of Analamazaotra (Périnet forest).

- ***Toxorhynchites* (*Afrorhynchus*) *grjebinei* Ribeiro, 2004 [** **190** **]**

Ribeiro, 2004 [190]

Endemic. Eggs undescribed. Larval habitats are *Ravenala*, as shown on one of the two labels of the holotype [190]. Occurs in the Périnet region, and in the Sainte-Luce region, far south-east of Madagascar [190].

- ***Toxorhynchites* (*Afrorhynchus*) *lemuriae* Ribeiro, 2004 [** **190** **]**

Ribeiro, 2004 [190]

Endemic. Only described and known from holotype female, which was collected in the Manakara region of the eastern domain [190].

- ***Toxorhynchites* (*Afrorhynchus*) *madagascarensis* Ribeiro, 2004 [** **190** **]**

Ribeiro, 2004 [190]

Endemic. Eggs undescribed. The holotype male was obtained from larval rearing after collection in *Ravenala*. Known only from the Taolagnaro area, in the eastern domain [67].

- ***Toxorhynchites* (*Afrorhynchus*) *pauliani* (Doucet, 1951) [** **67** **]**

Doucet, 1951 [67]

Endemic. Only known from the holotype male, which was collected by Doucet from an outdoor area (in leaf of *Ravenala*) in Vangaindrano city, in 1950 [67].

### 2.14 Genus *Uranotaenia* Lynch Arribálzaga, 1891

Genus *Uranotaenia* is the only genus of Culicidae belonging to the tribe Uranotaeniini. This genus is subdivided into two subgenera and includes 267 species in the world: the subgenus *Pseudoficalbia* (146 species) and the subgenus *Uranotaenia* (121 species) [119]. The genus *Uranotaenia* occurs on all continents, with the exception of the Pacific Ocean islands and Antarctica. This genus is particularly well represented in the Afrotropical and Oriental regions. In Madagascar, a comprehensive and complete systematic revision was carried out on the genus *Uranotaenia* [51]. With this revision, the genus *Uranotaenia* is the best represented and probably the best known genus, regarding the number of species in Madagascar. Among 73 Malagasy species belonging to this genus, 65 species are endemic, and four species occur only in Madagascar and in the Comoros archipelago. Although we have little information on the host preferences and behavior of adults, we know that these mosquitoes prefer to feed on cold-blooded animals (reptiles, amphibians), and nevertheless are probably involved in disease transmission.

#### 2.14.1 Subgenus *Pseudoficalbia* Theobald, 1912 [221]

All Malagasy species (*n* = 52) of this subgenus are endemic. This study did not include *Ur. comorensis*, *Ur. fusca*, *Ur. mashonaensis*, *Ur. nepenthes*, *Ur. ornate*, *Ur. pandani*, and *Ur. shillitonis* that were reported to be present on the island by WRBU [244]. The larval habitats are always associated with small breeding sites and phytotelmata.

- ***Uranotaenia* (*Pseudoficalbia*) *albimanus* da Cunha Ramos & Brunhes, 2004** [51]

da Cunha Ramos & Brunhes, 2004 [51]

Endemic. Egg and larval stages undescribed. Belongs to the Annulata section (Lavieri Group). Larval habitats are leaf axils of *Ravenala*. Collected only once and known only from the type locality (coastal forests of the Manakara region), in the eastern domain [51].

- ***Uranotaenia* (*Pseudoficalbia*) *albinotata* da Cunha Ramos & Brunhes, 2004** [51]

da Cunha Ramos & Brunhes, 2004 [51]

Endemic. Eggs undescribed. Belongs to section and group Shillitonis. Larval habitats are cut bamboo. Occurs only in the Manakara region, in the eastern domain.

- ***Uranotaenia* (*Pseudoficalbia*) *ambodimanga* da Cunha Ramos & Brunhes, 2004** [51]

da Cunha Ramos & Brunhes, 2004 [51]

Endemic. Eggs, larval and pupal stages undescribed. Belongs to section Annulata (Lavieri Group). Larval habitats are dried bamboo. Collected only once and known only from the type locality (Ambodimanga, Moramanga region) [51].

- ***Uranotaenia* (*Pseudoficalbia*) *antalahaensis* da Cunha Ramos & Brunhes, 2004** [51]

da Cunha Ramos & Brunhes, 2004 [51]

Endemic. Eggs, larval stages and adult male undescribed. Belongs to section Spinosa. Larval habitats are rotten *Ravenala* trunks containing plant organic matter. Collected on one occasion in the Masoala National Park, in the eastern domain [51].

- ***Uranotaenia* (*Pseudoficalbia*) *apicosquamata* da Cunha Ramos & Brunhes, 2004** [51]

da Cunha Ramos & Brunhes, 2004 [51]

Endemic. Eggs undescribed. Belongs to section Annulata (Lavieri Group). Larval habitats are sectioned *Ravenala* trunks, bamboo, mango tree trunks, and rock holes. Occurs in the eastern and western domains and in the Sambirano area (Nosy Be) [51].

- ***Uranotaenia* (*Pseudoficalbia*) *bambusicola* da Cunha Ramos & Brunhes, 2004** [51]

da Cunha Ramos & Brunhes, 2004 [51]

Endemic. Eggs and adult male undescribed. Belongs to section and group Shillitonis. Larval habitats are cut bamboo. Occurs in the eastern domain, at altitudes around 1000 m.

- ***Uranotaenia* (*Pseudoficalbia*) *belkini* Grjebine, 1979** [109]

Grjebine, 1979 [109]

Endemic. Eggs undescribed. Belongs to section and group Shillitonis. Larval habitats are *Nepenthes madagascariensis* pitchers. Occurs on the south-eastern coast of Madagascar from Manakara to Taolagnaro.

- ***Uranotaenia* (*Pseudoficalbia*) *bicincta* da Cunha Ramos & Brunhes, 2004** [51]

da Cunha Ramos & Brunhes, 2004 [51]

Endemic. Eggs and adult female undescribed. Belongs to section Shillitonis, sole representative of the Bicincta Group. Larval habitats are cut bamboo. Occurs in the Sambirano area (Nosy Be) and in the Antalaha region of the eastern domain [51].

- ***Uranotaenia* (*Pseudoficalbia*) *bifasciata* da Cunha Ramos & Brunhes, 2004** [51]

da Cunha Ramos & Brunhes, 2004 [51]

Endemic. Eggs and adult male undescribed. Belongs to section Annulata (Lavieri Group). Larval habitats are crab holes. Collected only once and known only from the type locality (Nosy Mangabe, in Antongil Bay in the eastern domain) [51].

- ***Uranotaenia* (*Pseudoficalbia*) *bosseri* Grjebine, 1979** [109]

Grjebine, 1979 [109]

Endemic. Eggs undescribed. Belongs to section Nigripes. Larval habitats are *Nepenthes madagascariensis* pitchers [109]. Occurs from the Manakara region to the Amboasary region, from the eastern domain to the southern domain boundary [51].

- ***Uranotaenia* (*Pseudoficalbia*) *boussesi* da Cunha Ramos & Brunhes, 2004** [51]

da Cunha Ramos & Brunhes, 2004 [51]

Endemic. Eggs, pupal and adult stages undescribed. Larval stages are morphologically close to those of *Uranotaenia ravenalicola*. Belongs to section Nigripes. Larval habitats are leaf axils of *Ravenala*. Collected only once and known only from the type locality (Ampasinalotra, in the Brickaville region of the eastern domain) [51].

- ***Uranotaenia* (*Pseudoficalbia*) *breviseta* da Cunha Ramos & Brunhes, 2004** [51]

da Cunha Ramos & Brunhes, 2004 [51]

Endemic. Eggs, larval stages and adult female undescribed. Belongs to section Annulata (Lavieri Group). Description of larva unknown. The larvae develop in *Ravenala* trunks. This species was collected only once and is known only from the type locality (coastal forest of Manakara of the eastern domain) [51].

- ***Uranotaenia* (*Pseudoficalbia*) *brumpti* Doucet, 1951** [67]

Doucet, 1951 [67]

Endemic. Eggs undescribed. Belongs to section Nigripes. Larval habitats are dry dead leaves of *Ravenala*, clear waters [67], puddles on tree trunks, banana leaves, and cut bamboo [51]. Occurs in the eastern domain [51], from sea level up to 1000 m.

- ***Uranotaenia* (*Pseudoficalbia*) *brunhesi* Grjebine, 1979** [109]

Grjebine, 1979 [109]

Endemic. Eggs undescribed. Belongs to section and group Shillitonis. Larval habitats are *Nepenthes madagascarensis* pitchers, in the Taolagnaro region of the far south-east of Madagascar [50, 109].

- ***Uranotaenia* (*Pseudoficalbia*) *cachani* (Doucet, 1950)** [65]

Doucet, 1950 [65]

Endemic. Eggs undescribed. Belongs to section Nigripes. Larval stages were formerly described as *Aedes* (*Skusea*) *cachani* by Doucet [65], classified in the genus *Uranotaenia* in 1955 by Mattingly & Brown [156]. Larval habitats are only the leaf axils of *Typhonodorum lindleyanum*. Occurs in the eastern and central domains [51].

- ***Uranotaenia* (*Pseudoficalbia*) *carcinicola* da Cunha Ramos & Brunhes, 2004** [51]

da Cunha Ramos & Brunhes, 2004 [51]

Endemic. Eggs undescribed. Belongs to section Annulata, and sole representative of the Annulata Group. Larval habitats are crab holes. Collected only once and known only from the type locality (Nosy Mangabe in Antongil Bay).

- ***Uranotaenia* (*Pseudoficalbia*) *combesi* Doucet, 1950** [65]

Doucet, 1950 [65]

Endemic. Eggs undescribed. Belongs to section Nigripes. Larval habitats are rock holes, pools near rice fields [65], bamboos resting on the beach, and basin cement. Occurs in the eastern, western, central, and southern domains [51, 65].

- ***Uranotaenia* (*Pseudoficalbia*) *contrastata* da Cunha Ramos & Brunhes, 2004** [51]

da Cunha Ramos & Brunhes, 2004 [51]

Endemic. Eggs, larval stages and adult male undescribed. Belongs to section and group Shillitonis. Larval habitats are cut bamboo. Collected only once and known only from the type locality (Ambodimanga village, in Moramanga district, at altitudes of about 400 to 500 m) [51].

- ***Uranotaenia* (*Pseudoficalbia*) *cornuta* da Cunha Ramos & Brunhes, 2004** [51]

da Cunha Ramos & Brunhes, 2004 [51]

Endemic. Eggs and adult male undescribed. This species belongs to section Spinosa. Larval habitats are leaf axils of *Pandanus*, exposed to the sun or located in forested areas. Occurs in the Lakato region (Moramanga district) and in the locality near the Bay of Saint-Luce (Taolagnaro region) of the eastern domain [51].

- ***Uranotaenia* (*Pseudoficalbia*) *damasei* Grjebine, 1979** [109]

Grjebine, 1979 [109]

Endemic. Eggs and adult stages undescribed. Belongs to section and group Shillitonis. Larval habitats are *Nepenthes madagascariensis* pitchers. Occurs on the south-eastern coast: from Manakara to Sainte-Luce [50, 109].

- ***Uranotaenia* (*Pseudoficalbia*) *donai* da Cunha Ramos & Brunhes, 2004** [51]

da Cunha Ramos & Brunhes, 2004 [51]

Endemic. Eggs, pupal and adult stages undescribed. Belongs to section and group Shillitonis. Larval habitats are *Nepenthes madagascariensis* pitchers, often hidden in sphagnum. Present only in the Taolagnaro region of the eastern domain [51].

- ***Uranotaenia* (*Pseudoficalbia*) *douceti* Grjebine, 1953** [103]

Grjebine, 1953 [103]

Endemic. Eggs undescribed. This species belongs to section Nigripes. Larval habitats are leaf axils of *Typhonodorum lindleyanum* and *Pandanus* [103, 182]. Occurs in the Sambirano area, western and eastern domains. Widely distributed at altitudes below 100 m asl [51, 103].

- ***Uranotaenia* (*Pseudoficalbia*) *fulgens* da Cunha Ramos & Brunhes, 2004** [51]

da Cunha Ramos & Brunhes, 2004 [51]

Endemic. Egg, pupal stages and adult male undescribed. Belongs to section and group Shillitonis. Larval habitats are cut bamboo. Occurs in the Moramanga region of the eastern domain [51].

- ***Uranotaenia* (*Pseudoficalbia*) *grenieri* Doucet, 1951** [67]

Doucet, 1951 [67]

Endemic. Eggs undescribed. Belongs to section Nigripes. Morphologically close to *Ur. ornata* Theobald, which is absent from Madagascar. Larval habitats are dead leaves of *Ravenala* [67], leaf axils of *Typhonodorum* and *Pandanus*, gutters, mushrooms horns, rock holes, fallen tree trunks, puddles, and streams [51]. Occurs in the central and eastern domains. Abundant from eastern seaboard up to 1200 m asl [51].

- ***Uranotaenia* (*Pseudoficalbia*) *grjebinei* da Cunha Ramos & Brunhes, 2004** [51]

da Cunha Ramos & Brunhes, 2004 [51]

Endemic. Eggs undescribed. Belongs to section Spinosa. Larval habitats are leaf axils of *Pandanus*. Occurs in the eastern domain [51].

- ***Uranotaenia* (*Pseudoficalbia*) *haddowi* da Cunha Ramos & Brunhes, 2004** [51]

da Cunha Ramos & Brunhes, 2004 [51]

Endemic. Eggs undescribed. Belongs to section Annulata (Lavieri Group). Larval habitats are mango tree trunks, Kapok trees, and *Ravenala*. Occurs in the Sambirano area (Nosy Be), northern, western, and eastern domains, always on coastal plains.

- ***Uranotaenia* (*Pseudoficalbia*) *hirsuta* Boussès & Brunhes, 2013** [14]

Boussès & Brunhes, 2013 [14]

Endemic. Eggs, pupal and adult stages undescribed. Belongs to section Annulata (Lavieri Group). Larval habitats are rock holes. Collected only once and known only from the type locality (Ambovanomby forest, in the Namoroka reserve of Mahajanga Province).

- ***Uranotaenia* (*Pseudoficalbia*) *hervyi* da Cunha Ramos & Brunhes, 2004** [51]

da Cunha Ramos & Brunhes, 2004 [51]

Endemic. Eggs undescribed. Belongs to section and group Shillitonis. Larval habitats are water retained in cut or broken bamboo. Occurs in the Moramanga region of the eastern domain [51].

- ***Uranotaenia* (*Pseudoficalbia*) *kraussi* Grjebine, 1953** [103]

Grjebine, 1953 [103]

Endemic. Eggs undescribed. Belongs to section Nigripes. Larval habitats are leaf axils of Taro [103] and *Typhonodorum* sp., banana leaves, bamboo, and mushroom caps [51]. Carnivorous larvae, which feed on mosquito larvae of the same or other species. Rare species, but widely distributed throughout Montagne d’Ambre in the northern domain, Mandraka in Antananarivo province, Ikongo of Fianarantsoa province in the central domain, and Andasibe forest in the eastern domain [51].

- ***Uranotaenia* (*Pseudoficalbia*) *laffosseae* da Cunha Ramos & Brunhes, 2004** [51]

da Cunha Ramos & Brunhes, 2004 [51]

Endemic. Eggs undescribed. Belongs to section Nigripes. Larval habitats are cut bamboo and tree holes [51] and axils of *Typhonodorum* in Mayotte [145]. This species is present in Madagascar and Mayotte [145]. In Madagascar, occurs in the eastern domain and in the Montagne d’Ambre of the northern domain [51].

- ***Uranotaenia* (*Pseudoficalbia*) *lavieri* Doucet, 1950** [65]

Doucet, 1950 [65]

Endemic. Eggs and adult stages undescribed. Belongs to section Annulata (representative of the nominal group). Larval habitats are cut bamboo [66] and tree holes [51]. Occurs in the Sambirano area (Nosy Be, Nosy Komba) [90], in rainforests of the eastern [66] and northern domains [51].

- ***Uranotaenia* (*Pseudoficalbia*) *legoffi* da Cunha Ramos & Brunhes, 2004** [51]

da Cunha Ramos & Brunhes, 2004 [51]

Endemic. Eggs undescribed. Belongs to section Spinosa. Larval habitats are bamboo and leaf axils of *Pandanus* on the Pangalana canal. Occurs from the Brickaville region to the margins of the eastern domain, up to 500 m asl [51].

- ***Uranotaenia* (*Pseudoficalbia*) *longitubus* da Cunha Ramos & Brunhes, 2004** [51]

da Cunha Ramos & Brunhes, 2004 [51]

Endemic. Eggs, pupal and adult stages undescribed. Belongs to section Annulata (Lavieri Group). Larval habitats are cut bamboo. Collected only once and known only from the type locality (Masoala island in the eastern domain) [51].

- ***Uranotaenia* (*Pseudoficalbia*) *lousthei* Boussès & Brunhes, 2013** [14]

Boussès & Brunhes, 2013 [14]

Endemic. Only the larval stages were described [14]. Belongs to section Spinosa [51]. Larval habitats are leaf axils of *Pandanus* along the beachfront. Collected only once and known only from the type locality (Ambila-Lemaitso, Toamasina province of the eastern domain).

- ***Uranotaenia* (*Pseudoficalbia*) *madagascarensis* da Cunha Ramos & Brunhes, 2004** [51]

da Cunha Ramos & Brunhes, 2004 [51]

Endemic. Eggs undescribed. Belongs to section and group Shillitonis. Larval habitats are cut bamboo. Occurs in the eastern domain, from sea level up to 1000 m altitude [51].

- ***Uranotaenia* (*Pseudoficalbia*) *manakaraensis* da Cunha Ramos & Brunhes, 2004** [51]

da Cunha Ramos & Brunhes, 2004 [51]

Endemic. Eggs and larval stages undescribed. Belongs to section Annulata (Lavieri Group). Larval habitats are *Ravenala* trunks. Occurs only in the Manakara region, in the eastern domain [51].

- ***Uranotaenia* (*Pseudoficalbia*) *nigricephala* da Cunha Ramos & Brunhes, 2004** [51]

da Cunha Ramos & Brunhes, 2004 [51]

Endemic. Eggs undescribed. Belongs to section Nigripes. Larval habitats are leaf axils of *Typhonodorum* and *Pandanus* sp. Occurs in the eastern domain.

- ***Uranotaenia* (*Pseudoficalbia*) *nigripleura* da Cunha Ramos & Brunhes, 2004** [51]

da Cunha Ramos & Brunhes, 2004 [51]

Endemic. Eggs and larval stages undescribed. Belongs to section Spinosa. Larval habitats are leaf axils of *Ravenala* along the beachfront of the eastern domain [51].

- ***Uranotaenia* (*Pseudoficalbia*) *pallidipleura* da Cunha Ramos & Brunhes, 2004** [51]

da Cunha Ramos & Brunhes, 2004 [51]

Endemic. Further description of egg and larval stages will perhaps help to distinguish *Ur. pallidipleura* from *Ur. donai* [51]. Belongs to section and group Shillitonis. Larval habitats are *Nepenthes madagascariensis* pitchers. Occurs on the south-eastern coast of Madagascar.

- ***Uranotaenia* (*Pseudoficalbia*) *pauliani* Doucet, 1949** [64]

Doucet, 1949 [64]

Endemic. Only the larval stages were described [64]. Belongs to section Nigripes. Formerly synonymized with *Ur. nepenthes* and *Ur. pandani* which are endemic to the Seychelles archipelago [124, 156]. Morphologically close to *Ur. ornate* which is absent from Madagascar [64]. Only type series of larval stages were caught to date. Larval habitats are dried leaf rachis of *Neodypsis* on the ground. Collected only once. Known only from the type locality (Ambohiby, Tsiroanomandidy region in the central domain) [64].

- ***Uranotaenia* (*Pseudoficalbia*) *pilosa* da Cunha Ramos & Brunhes, 2004** [51]

da Cunha Ramos & Brunhes, 2004 [51]

Endemic. Eggs and adult female undescribed. Belongs to section Spinosa. Larval habitats are leaf axils of *Pandanus* throughout the beachfront to the mountainous regions of the eastern slopes. Occurs in the central and eastern domains.

- ***Uranotaenia* (*Pseudoficalbia*) *pseudoalbimanus* da Cunha Ramos & Brunhes, 2004** [51]

da Cunha Ramos & Brunhes, 2004 [51]

Endemic. Eggs undescribed. Belongs to section Annulata (Lavieri Group). Larval habitats are *Ravenala* trunks and cut bamboo. Occurs in the central and eastern domains [51]. Frequent in humid forest areas or areas formerly occupied by forest.

- ***Uranotaenia* (*Pseudoficalbia*) *pseudoshillitonis* da Cunha Ramos & Brunhes, 2004** [51]

da Cunha Ramos & Brunhes, 2004 [51]

Endemic. Eggs undescribed. Belongs to section and group Shillitonis. Larval habitats are cut bamboo. Occurs in the central and eastern domains [51].

- ***Uranotaenia* (*Pseudoficalbia*) *ravenalaphila* Boussès & Brunhes, 2013** [14]

Boussès & Brunhes, 2013 [14]

Endemic. Only the larval stages were described [14]. Belongs to section Annulata. Larval habitats are cut trunks of *Ravenala madagascariensis* containing brown water and rotting vegetable matter. Its larval stages are usually found in association with *Uranotaenia haddowi*, *Ur. manakaraensis*, *Ur. albimanus*, and *Ur. breviseta*. Collected only once and known only from the type locality (Ifaho, Manakara region of the eastern domain).

- ***Uranotaenia* (*Pseudoficalbia*) *ravenalicola* da Cunha Ramos & Brunhes, 2004** [51]

da Cunha Ramos & Brunhes, 2004 [51]

Endemic. Eggs undescribed. Belongs to section Nigripes. Morphologically close to *Ur. boussesi*. Larval habitats are leaf axils of *Ravenala*. Occurs in the eastern domain, from sea level up to about 1000 m asl.

- ***Uranotaenia* (*Pseudoficalbia*) *scutostriata* da Cunha Ramos & Brunhes, 2004** [51]

da Cunha Ramos & Brunhes, 2004 [51]

Endemic. Eggs undescribed. Belongs to section Annulata (Lavieri Group). Larval habitat is bamboo forest. Collected only once and known only from the type locality (Ambodimanga, Moramanga region of the eastern domain) [51].

- ***Uranotaenia* (*Pseudoficalbia*) *spinitubus* da Cunha Ramos & Brunhes, 2004** [51]

da Cunha Ramos & Brunhes, 2004 [51]

Endemic. Only the larval stages were described [51]. Belongs to section Annulata (Lavieri Group). Larval habitats are tree holes. Occurs in the northern and eastern domains [51].

- ***Uranotaenia* (*Pseudoficalbia*) *spinosa* da Cunha Ramos & Brunhes, 2004** [51]

da Cunha Ramos & Brunhes, 2004 [51]

Endemic. Eggs and adult male undescribed. Belongs to section Spinosa. Larval habitats are leaf axils of *Pandanus* on the beachfront of the eastern domain [51]

- ***Uranotaenia* (*Pseudoficalbia*) *spiraculata* da Cunha Ramos & Brunhes, 2004** [51]

da Cunha Ramos & Brunhes, 2004 [51]

Endemic. Eggs undescribed. Belongs to section and group Shillitonis. Larval habitats are cut bamboo. Occurs in the eastern domain, and is particularly abundant in the eastern forest (Beforona, Périnet), at an altitude between 500 and 1000 m.

- ***Uranotaenia* (*Pseudoficalbia*) *tricolor* da Cunha Ramos & Brunhes, 2004** [51]

da Cunha Ramos & Brunhes, 2004 [51]

Endemic. Only the larval stages were described [51]. Belongs to section Spinosa. Larval habitats are leaf axils of *Pandanus* along the beachfront of the eastern coast.

- ***Uranotaenia* (*Pseudoficalbia*) *tridentata* da Cunha Ramos & Brunhes, 2004** [51]

da Cunha Ramos & Brunhes, 2004 [51]

Endemic. Only the larval stages were described [51]. Belongs to section and group Shillitonis. Larval habitats are bamboo. Occurs in the eastern domain, between 800 and 1000 m asl.

- ***Uranotaenia* (*Pseudoficalbia*) *tsaratananae* Doucet, 1950** [65]

Doucet, 1950 [65]

Endemic. Eggs undescribed. Belongs to section Nigripes. Larval habitats are tree holes [65], palm tree forming a gutter, coconuts, and the leaf axils of *Pandanus* [51]. Occurs in high altitude forests of the eastern and northern domains [51], between 700 and 1700 m asl.

#### 2.14.2 Subgenus *Uranotaenia* Lynch Arribálzaga, 1891

This subgenus includes 21 species in Madagascar. Among them, *Ur. alba* was reported to be absent from the island by WRBU [244]. Fourteen species are endemic to the island, three are found in Madagascar and in the Comoros archipelago, and only four are African-Malagasy species.

- ***Uranotaenia* (*Uranotaenia*) *alba* Theobald, 1901**

Doucet, 1951 [67]

Eggs undescribed. Belongs to section Alba (Alba Group). Larval habitats are muddy rice fields. Occurs in the eastern domain [51, 67].

- ***Uranotaenia* (*Uranotaenia*) *alboabdominalis* Theobald, 1910** [220]

Doucet, 1951 [67]

Eggs undescribed. Belongs to section Alba (Alba Group). Larval habitats are swamps, puddles under forest cover, and ponds [67]. Occurs mainly in the eastern domain and in the Mahajanga region of the western domain [51].

- ***Uranotaenia* (*Uranotaenia*) *albocephala* da Cunha Ramos & Brunhes, 2004** [51]

da Cunha Ramos & Brunhes, 2004 [51]

Endemic. Eggs and adult male remain to be described. Belongs to section and group Anopheloides. Larval habitats are tree holes with water containing organic matter. Collected only once and known only from the type locality (Masoala peninsula, in the eastern domain) [51].

- ***Uranotaenia* (*Uranotaenia*) *andavakae* Doucet, 1950** [65]

Doucet, 1950 [65]

Endemic to Madagascar and the Comoros archipelago (Mayotte) [65]. Eggs undescribed. Belongs to section Anopheloides (Neireti Group). Larval habitats are cool and clear waters such as fountains, ditches with sphagnum, puddles, flooded meadows, rice fields, forest ponds, rock holes, and trickle of water along a stream [51, 65]. Occurs in the eastern domain, in mountainous areas of the western, eastern, and central domains [51], and can reach about 1800 m asl.

- ***Uranotaenia* (*Uranotaenia*) *anopheloides* Brunhes & Razafindrasolo, 1975** [31]

Brunhes & Razafindrasolo, 1975 [31]

Endemic to Madagascar and to the Comoros archipelago (Mayotte) [31]. Eggs undescribed. Belongs to section Anopheloides. Larval stages are characterized by their bodies floating parallel to the surface of the water like those of *Anopheles*. Larval habitats are tree holes (mango tree, kapok tree, and *Ravenala*) [51]. Occurs in the western, central, and eastern domains, seems more abundant in the warmer regions of the western domain. Eggs are resistant to desiccation [51].

- ***Uranotaenia* (*Uranotaenia*) *argentipleura* da Cunha Ramos & Brunhes, 2004** [51]

da Cunha Ramos & Brunhes, 2004 [51]

Endemic. Egg, larval and pupal stages undescribed. Belongs to section Caeruleocephala (Madagascarica Group). Larval habitats are grassy marshes. Occurs in the Sambirano area (Nosy Be) and the dry regions of the western and southern domains.

- ***Uranotaenia* (*Uranotaenia*) *balfouri* Theobald, 1904**

Doucet, 1949 [63]

Eggs undescribed. Belongs to section Alba (Balfouri Group). Morphologically close to *Uranotaenia hebrardi*. In Madagascar, larval habitats are canals containing iron hydroxide, rice fields, pools, marshes [63], grassy holes [103], muddy water under forest cover, swamps, and ponds [51]. Occurs in the Sambirano area (Nosy Be), in the western, eastern, and central domains. This species is found in Madagascar from sea level up to 1700 m asl [51]. Adult biology unknown. In Africa, feeds mainly on amphibians and cattle in Kenya [13].

- ***Uranotaenia* (*Uranotaenia*) *bidentata* da Cunha Ramos & Brunhes, 2004** [51]

da Cunha Ramos & Brunhes, 2004 [51]

Endemic. Eggs, pupal and adult stages undescribed. Belongs to section Chorleyi (Hamoni Group). Biology unknown. Occurs in the Manambolosy area of Toamasina province and in the Ampatsinakoho area of Fianarantsoa province. Seems to be widely distributed throughout the eastern domain (from the Vangaindrano region to the Mananara region) at lower altitudes.

- ***Uranotaenia* (*Uranotaenia*) *connali* Edwards, 1912** [73]

da Cunha Ramos & Brunhes, 2004 [51]

Eggs, larval and pupal stages undescribed. Belongs to section Alba (Bilineata Group). Biology unknown. In Madagascar, captured once and occurs on the Masoala peninsula in the eastern domain [51].

- ***Uranotaenia* (*Uranotaenia*) *dumonti* Doucet, 1949** [64]

Doucet, 1949 [64]

Endemic. Only the larval stages were described [64]. Belongs to section and group Dumonti. Larval habitats are water holes and reed beds. Collected only once and known only from the type locality (Ambohiby mountain stream, at 1633 m asl, in the Tsiroanomandidy region of the central domain) [51, 64].

- ***Uranotaenia* (*Uranotaenia*) *geniculata* da Cunha Ramos & Brunhes, 2004** [51]

da Cunha Ramos & Brunhes, 2004 [51]

Endemic. Eggs undescribed. Belongs to section Caeruleocephala (Madagascarica Group). Larval habitats are tree holes containing clear water and cut bamboo. Occurs only on the Masoala peninsula in the eastern domain [51].

- ***Uranotaenia* (*Uranotaenia*) *grassei* da Cunha Ramos & Brunhes, 2004** [51]

da Cunha Ramos & Brunhes, 2004 [51]

Endemic. Eggs and adult male undescribed. Belongs to section and group Dumonti. Larval habitats are streams of the relict primary rainforest and in holes containing clean and cool water. Occurs in the eastern domain [51].

- ***Uranotaenia* (*Uranotaenia*) *hamoni* Grjebine, 1953** [103]

Grjebine, 1953 [103]

Endemic. Eggs undescribed. Originally described under the name of *Uranotaenia chorleyi* var. *hamoni* [103] and formally elevated to species rank by White [238]. Belongs to section Chorleyi (Hamoni Group). Larval habitats are forest ponds, rivers, holes containing clear and fresh water, rice fields surrounded by forest, and forest streams. Occurs in the central, eastern, and western domains and was mainly caught in mountainous areas, above 800 m asl [51].

- ***Uranotaenia* (*Uranotaenia*) *hebrardi* da Cunha Ramos & Brunhes, 2004** [51]

da Cunha Ramos & Brunhes, 2004 [51]

Endemic. Belongs to section Alba. Morphologically close to *Uranotaenia balfouri* (Balfouri Group). Larval habitats are ponds, rice fields, and marshes. Occurs in the eastern, western, and central domains. Caught only as larvae and on five capture occasions in the Amborompotsy [191] and Manakara areas of Fianarantsoa province, the Marvoay area of Mahajanga province, and the Ambodimanga and Moramanga areas of Toamasina province [51]

- ***Uranotaenia* (*Uranotaenia*) *joucouri* da Cunha Ramos & Brunhes, 2004** [51]

da Cunha Ramos & Brunhes, 2004 [51]

Endemic. Eggs, pupal and adult stages undescribed. Belongs to Alba section and Group. Larval habitats are unknown. Collected only once and known only from the type locality (Foulpointe, in the eastern domain) [51].

- ***Uranotaenia* (*Uranotaenia*) *lebiedi* da Cunha Ramos & Brunhes, 2004** [51]

da Cunha Ramos & Brunhes, 2004 [51]

Endemic. Eggs, larval and pupal stages undescribed. Belongs to section and group Dumonti. Adult biology unknown. Occurs in the eastern domain, and was caught as adult stage, below 900 m altitude [51].

- ***Uranotaenia* (*Uranotaenia*) *madagascarica* da Cunha Ramos & Brunhes, 2004** [51]

da Cunha Ramos & Brunhes, 2004 [51]

Endemic. Eggs undescribed. Belongs to section Caeruleocephala. Larval habitats are holes containing plant debris and iron hydroxide. Occurs in the Ampatsinakoho area (Fianaranstsoa province) and in forested areas of the eastern domain: Mandena forest (Taolagnaro), Masoala National Park (Antsiranana province), and Andasibe National Park (Toamasina province) [51].

- ***Uranotaenia* (*Uranotaenia*) *mayottensis* Brunhes, 1977** [25]

da Cunha Ramos & Brunhes, 2004 [51]

Endemic to Madagascar and the Comoros archipelago (Mayotte). Eggs undescribed. Belongs to Dumonti section. Larval habitats are fresh water, rivers, small forest streams [51], slow-flowing water, rock holes, and water ponds [145]. Present in Madagascar and on Mayotte. Occurs in the eastern, western, and southern domains, in Malagasy National Parks (Masoala, Bemaraha, Andasibe, and Ranomafana parks) and was caught in the Isaka area of Toliara province [51].

- ***Uranotaenia* (*Uranotaenia*) *moramangae* da Cunha Ramos & Brunhes, 2004** [51]

da Cunha Ramos & Brunhes, 2004 [51]

Endemic. Eggs undescribed. This species belongs to section Chorleyi (Hamoni Group). Larval habitats are forest puddles, streams containing sphagnum, fresh and acidic water, cattle hoof prints, ditches, and grassy swamps. Occurs in the central, eastern, and southern domains [51] and is distributed between 450 m (Beraketa, Toliary province) and 1800 m asl (Ankaratra massif).

- ***Uranotaenia* (*Uranotaenia*) *neireti* Edwards, 1920** [75]

Edwards, 1920 [75]

Endemic. Eggs undescribed. Belongs to Anopheloides section. Larval habitats are hoof prints, lakes, and swamps areas. Present in the central and eastern domains and always between 900 and 2000 m asl [51].

- ***Uranotaenia* (*Uranotaenia*) *roberti* da Cunha Ramos & Brunhes, 2004** [51]

da Cunha Ramos & Brunhes, 2004 [51]

Endemic. Eggs, pupal and adult stages undescribed. Belongs to Chorleyi section (Hamoni Group). Larval habitats are slow-flowing water of small forest streams of primary rainforest. Collected only once and known only from the type locality (Lakato area, above 1000 m asl of the eastern domain) [51].

## 3. Discussion

The main remarkable characteristic of Malagasy mosquito fauna is the high biodiversity with 138 (of 235) endemic species (58.7%). This pattern is not only specific to mosquitoes since Madagascar has one of the highest rates of endemicity in the world, due to its insularity with long geographic isolation since the migration of the Indo-Malagasy subcontinent 155–120 million years ago. The isolation of the island dates from the Upper Cretaceous (83 million years). Paulian [173] highlighted this endemism rate in many vertebrate and invertebrate groups. The endemism rate is about 92–100% for terrestrial animals and 52–60% for flying animals (bats and birds) [100]. As an example, 75% (97/130) of *Drosophila* species [135], all of the 40 species of scorpions [149], and 91% (379/418) of ant species [84] are endemic to Madagascar.

Among other hematophagous arthropods other than mosquitoes, the endemism rate is 92% (11/12) in sand flies (Vincent Robert, pers. comm.), 78.2% (36/46 species) in fleas (Sébastien Boyer, unpublished observation), and 79% (27/34) in ticks [226].

### 3.1 Diversity

In total 235 mosquitoes species are currently recorded in Madagascar. These 235 species belong to 14 genera: *Aedeomyia* (3 species), *Aedes* (35 species), *Anopheles* (26 species), *Coquillettida* (3 species), *Culex* (at least 50 species), *Eretmapodites* (4 species), *Ficalbia* (2 species), *Hodgesia* (at least one species), *Lutzia* (one species), *Mansonia* (2 species), *Mimomyia* (22 species), *Orthopodomyia* (8 species), *Toxorhynchites* (6 species), and *Uranotaenia* (73 species). This number of 235 species is considerably higher than that given in the previous checklist published in 2003 (i.e. 178 species) [70] (Tables 1 and 2). This main difference is primarily due to the inclusion of an additional collection of *Uranotaenia* [14, 51], *Toxorhynchites* [190], *Aedeomyia* [33], and *Aedes* belonging to the subgenus *Neomelaniconion* [143]. However, this number is moderately lower than that given in Internet references such as WRBU (253 species) [244] and Arim (245 species) [5], which include an enlarged area of several islands in the Mozambique Channel.

**Table 1:** Distribution of restricted endemic species for each Malagasy bioclimatic domain.

	Endemic East	Endemic West	Endemic Sambirano	Endemic North	Endemic South	Endemic Centre	Endemic Mountain	Endemic Madagascar†	Endemic Madagascar and Comoros§	Number of species#
*Aedeomyia*	1	0	0	0	0	0	0	2	0	3
*Aedes*	7	0	0	1	0	0	0	18	2	35
*Anopheles*	2	0	0	0	0	1	1	11	1	26
*Coquillettidia*	0	0	0	0	0	0	0	2	0	3
*Culex*	3	0	0	0	0	4	0	9	3	50*
*Eretmapodites*	0	0	0	0	0	0	0	0	0	4
*Ficalbia*	0	0	0	0	0	0	0	0	0	2
*Hodgesia*	?	0	0	0	0	0	0	?	0	≥1
*Lutzia*	0	0	0	0	0	0	0	0	0	1
*Mansonia*	0	0	0	0	0	0	0	0	0	2
*Mimomyia*	12	0	1	0	0	2	0	17	0	22
*Orthopodomyia*	3	0	0	1	0	1	1	8	0	8
*Toxorhynchites*	6	0	0	0	0	0	0	6	0	6
*Uranotaenia*	42	1	0	0	0	2	2	65	4	73
Total	76	1	1	2	0	10	4**	138	10	235

† Endemic mosquitoes restricted to Madagascar.

§ Endemic mosquitoes in Madagascar and the Comoros archipelago.

# Species described and/or clearly identified.

* The species *Culex thalassius*, mentioned with doubt in Madagascar by Knight and Stone [132] and listed in Madagascar by the site WRBU (www.mosquitocatalog.org/, August 2014) [244], has not been recorded in this table; similarly the species belonging to the Rima group surveyed but remaining unidentified by Rhodain et al. [195] have not been recorded. *Cx. pipiens* and *Cx. quinquefasciatus* were counted as two separate species following the conclusion of Harbach [118].

** The four species of mosquitoes known only in mountains areas (altitude >1500 m.) are usually associated with the central bioclimatic domain.

**Table 2.** Summary information on the 235 mosquito species that occur in Madagascar.

			Biology of each species on Madagascar				Pathogens associated with each species	
Genus	Species	Unknown stages	Endemicity	Specific climatic domain	Host preferences	Larval habitats	Madagascar	Africa
*Anopheles*	*coustani*				GF	A, N	BABV£, PERV, RVFV, WNV, F	
*Anopheles*	*fuscicolor*		X			A, N	BABV£, PERV£, RVFV£, F	
*Anopheles*	*tenebrosus*			O, N, C, W		A, N		P
*Anopheles*	*arabiensis*				GF			
*Anopheles*	*brunnipes*			W, E		A, N	WNV	
*Anopheles*	*cydippis*					T, A, N		
*Anopheles*	*flavicosta*					A, N		V, F, P
*Anopheles*	*funestus*					A, N		V
*Anopheles*	*gambiae*				GF			
*Anopheles*	*grassei*		X	W, S		N	P	
*Anopheles*	*grenieri*	F M	X	E		A, N		
*Anopheles*	*griveaudi*	M L	X	C				
*Anopheles*	*lacani*	M	X	C, E		N		
*Anopheles*	*maculipalpis*				GF	A, N	WNV	F, P*
*Anopheles*	*mascarensis*		X°		GF	A, N	NGAV, F, P	
*Anopheles*	*merus*			W, S		N	P	
*Anopheles*	*milloti*		X			N		
*Anopheles*	*notleyi*		X	N, E		N		
*Anopheles*	*pauliani*		X		GF	A, N	RVFV£, WNV, F	
*Anopheles*	*pharoensis*					A, N		V, F, P*
*Anopheles*	*pretoriensis*				GF	A, N		V, P*
*Anopheles*	*radama*		X	O, N, W, E		N		
*Anopheles*	*ranci*		X	N, E		A, N		
*Anopheles*	*roubaudi*		X	E		N		
*Anopheles*	*rufipes*				GF	A, N		V, P°
*Anopheles*	*squamosus*				Z	A, N	ANDV, BTV, RVFV, F	V
*Aedeomyia*	*madagascarica*	F M	X	E, W	O		WNV	
*Aedeomyia*	*pauliani*	L	X	E		P		
*Aedeomyia*	*furfurea*			E, C, W		P		
*Aedes*	*albocephalus*			O, N, W, S, E	GF	N		
*Aedes*	*albodorsalis*	M L	X	W, E	A			
*Aedes*	*argenteopunctatus*			E, C	A	N	DBV	V, Nem
*Aedes*	*dalzieli*			W, S		N		V, Nem
*Aedes*	*domesticus*			E		N		V
*Aedes*	*durbanensis*			S, W		N		V
*Aedes*	*fowleri*			W, S, E, C		A, N		V, Set
*Aedes*	*masoalensis*	M L	X	E	A		MgV	
*Aedes*	*mathioti*	M L	X	E	A			
*Aedes*	*natronius*			S				V
*Aedes*	*fryeri*			W, S	A	N		V£
*Aedes*	*coulangesi*	L	X	W, N, S	A			
*Aedes*	*grassei*	L	X	E				
*Aedes*	*madagascarensis*	L	X	O, N, W, C, E	A		WNV	
*Aedes*	*sylvaticus*	F L	X	E				
*Aedes*	*tiptoni*		X		A	P		
*Aedes*	*vittatus*			O, W, C, E, S	A	A, N		V
*Aedes*	*scatophagoides*		S		N			
*Aedes*	*mucidus*			E				
*Aedes*	*albiradius*	M L	X	W, C, S				
*Aedes*	*belleci*		X	E		N		
*Aedes*	*circumluteolus*			W, E, C	A	N	WNV	V
*Aedes*	*fontenillei*	L	X	E		N		
*Aedes*	*nigropterum*	L	X	E				
*Aedes*	*sylvaticum*	L	X	N, E				
*Aedes*	*ambreensis*	M L	X	N	A		MMP 158 Virus	
*Aedes*	*cartroni*	L	X°	W, S, N, E	A	N	MgV	
*Aedes*	*lambrechti*			O, N		N		
*Aedes*	*moucheti*	F L	X	O, W, C, E, S		N		
*Aedes*	*aegypti*				A	T, P	BABV, MMP 158, WNV	V
*Aedes*	*albopictus*				A	T, P	BABV	V
*Aedes*	*brygooi*		X	O, N, W, S, C		P		
*Aedes*	*interruptus*	L	X	E, C		T		
*Aedes*	*monetus*		X°	N, W, S, E		P		
*Aedes*	*phillipi*		X	O, E, W, N, C		P		
*Coquillettidia*	*grandidieri*		X	W, E, C	GF	N	RVFV£	
*Coquillettidia*	*metallica*			W, E, C	A			V, Pµ
*Coquillettidia*	*rochei*	L	X	W, E, C	A			
*Culex*	*antennatus*				GF	A, N		V, F, Pµ
*Culex*	*argenteopunctatus*		X	C, E		N		
*Culex*	*carleti*		X°	O, E	A	P		
*Culex*	*comorensis*		X°	C, E		P, T, N		
*Culex*	*decens*				GF	P, A, N	BABV, WNV	V
*Culex*	*demeilloni*	M F	X	C				
*Culex*	*duttoni*			O, C		N		V
*Culex*	*grahamii*			C				
*Culex*	*guiarti*			E	A	N		V, Pµ
*Culex*	*neavei*			C				V, Pµ
*Culex*	*perfidiosus*			E		A, N		
*Culex*	*pipiens*			C, W	GF	T, A		V, Pµ
*Culex*	*quasiguiarti*			C, E, S				
*Culex*	*quinquefasciatus*				GF	T	WN, BABV, PERV, F	V, Pµ
*Culex*	*scottii*	L		C, E	A		WNV	
*Culex*	*simpsoni*			O, E	A	T, N	RVFV£	
*Culex*	*sitiens*			O, W, C				V
*Culex*	*striatipes*			E, C		A, N		
*Culex*	*theileri*			E				
*Culex*	*tritaeniorhynchus*				GF	A	MgV, WNV	V, Pµ§
*Culex*	*perfuscus*			O				V
*Culex*	*trifilatus*	L		W				
*Culex*	*univittatus*			W, S, E, C	Z	A	BABV£, MgV	V, F, Pµ
*Culex*	*ventrilloni*	L	X	C				
*Culex*	*watti*			O, E		N		
*Culex*	*vansomereni*			E			BABV£, RVFV£	V
*Culex*	*weschei*			C	A	N		V
*Culex*	*cinerellus*			O, E		P		
*Culex*	*cinereus*			O, W		P		V
*Culex*	*milloti*	M F	X	C		N		
*Culex*	*nebulosus*			O, W, E, C	A	P		V
*Culex*	*pandani*		X	E	A	P		
*Culex*	*subaequalis*	F		?				
*Culex*	*moucheti*			E				V
*Culex*	*brenguesi*		X	E		N		
*Culex*	*chauveti*		X°	E, C	A	N		
*Culex*	*horridus*			W, C		P		
*Culex*	*insignis*			O				
*Culex*	*kingianus*			W, E		P, N		
*Culex*	*rubinotus*			O, E		P		V
*Culex*	*sunyaniensis*			E		P, N		
*Culex*	*wigglesworthi*			E				
*Culex*	*salisburiensis/coursi*	M F	X	E		A		
*Culex*	*salisburiensis/salisburiensis*			C				
*Culex*	*seyrigi*		X	C		N		
*Culex*	*aurantapex*	M		E	A			
*Culex*	*annulioris*			E, C, S			RVFV£	
*Culex*	*bitaeniorhynchus*		W, E, C, S	GF	A, N		V, Pµ* §	
*Culex*	*giganteus*		X	C, E, W, S	GF	A, N		
*Culex*	*poicilipes*			W, C, E	GF	A, N		V, F, Pµ
*Eretmapodites*	*oedipodeios*	L		E				V
*Eretmapodites*	*plioleucus*	L		O, E				
*Eretmapodites*	*quinquevittatus*			A	P, N	MgV	V	
*Eretmapodites*	*subsimplicipes*			E	A$			V
*Ficalbia*	*uniformis*			E, C		N		
*Ficalbia*	*circumtestacea*			E, W				
*Hodgesia*	*Hodgesiasp*			E	A	N		
*Lutzia*	*tigripes*			O, W, E, C		T, A, N		V
*Mansonia*	*africana*			W	A			F, V
*Mansonia*	*uniformis*			W, S, E, C	GF	A, N	BABV, PERV, RVFV, WNV, F	V, Pµ
*Mimomyia*	*martinei*	M L	X	E				
*Mimomyia*	*mediolineata*			W, E	A	N		
*Mimomyia*	*aurata*		X	C, E	A	P		
*Mimomyia*	*bernardi*		X	E		P		
*Mimomyia*	*beytouti*		X	E		P		
*Mimomyia*	*brygooi*	F	X	E		P		
*Mimomyia*	*collessi*		X	E				
*Mimomyia*	*jeansottei*		X	E		P		
*Mimomyia*	*levicastilloi*	M F	X	E		P		
*Mimomyia*	*longicornis*		X	E		P		
*Mimomyia*	*marksae*		X	E		P		
*Mimomyia*	*mattinglyi*	F	X	E		P		
*Mimomyia*	*milloti*		X	C		P		
*Mimomyia*	*ramalai*	M	X	C		P		
*Mimomyia*	*roubaudi*		X	W, E		P		
*Mimomyia*	*spinosa*		X	E		P		
*Mimomyia*	*stellata*		X	E		P		
*Mimomyia*	*vansomerenae*	M F	X	O		P		
*Mimomyia*	*hispida*			C		A, N		
*Mimomyia*	*mimomyiaformis*		W, E		N			
*Mimomyia*	*plumosa*			O, E		N		
*Mimomyia*	*splendens*			W, E		N		
*Orthopodomyia*	*ambremontis*	F	X	N		P		
*Orthopodomyia*	*ankaratrensis*	M F	X	C		P		
*Orthopodomyia*	*fontenillei*		X	C, E		P		
*Orthopodomyia*	*milloti*		X	C, E		P		
*Orthopodomyia*	*rajaonariveloi*	M L	X	E				
*Orthopodomyia*	*ravaonjanaharyi*	X	N		P			
*Orthopodomyia*	*rodhaini*	L	X	E		P		
*Orthopodomyia*	*vernoni*		X	W, E		P, T		
*Toxorhynchites*	*brunhesi*		X	E				
*Toxorhynchites*	*fontenillei*	F L	X	E		P		
*Toxorhynchites*	*grjebinei*		X	E		P		
*Toxorhynchites*	*lemuriae*	M L	X	E		P		
*Toxorhynchites*	*madagascarensis*		X	E		P		
*Toxorhynchites*	*pauliani*	M L	X	E		P		
*Uranotaenia*	*albimanus*	L	X	E		P		
*Uranotaenia*	*albinotata*		X	E		P		
*Uranotaenia*	*ambodimanga*	L	X	E		P		
*Uranotaenia*	*antalahaensis*	M L	X	E		P		
*Uranotaenia*	*apicosquamata*	X	O, W, E		P, N			
*Uranotaenia*	*bambusicola*	M	X	E		P		
*Uranotaenia*	*belkini*		X	E		P		
*Uranotaenia*	*bicincta*	F	X	O, E		P		
*Uranotaenia*	*bifasciata*	M	X	E		N		
*Uranotaenia*	*bosseri*		X	E		P		
*Uranotaenia*	*boussesi*	M F	X	E		P		
*Uranotaenia*	*breviseta*	F L	X	E		P		
*Uranotaenia*	*brumpti*		X	E		P, N		
*Uranotaenia*	*brunhesi*		X	E		P		
*Uranotaenia*	*cachani*		X	C, E		P		
*Uranotaenia*	*carcinicola*		X	E		N		
*Uranotaenia*	*combesi*		X	W, E, C, S		T, P, N		
*Uranotaenia*	*contrastata*	M L	X	E		P		
*Uranotaenia*	*cornuta*	M	X	E		P		
*Uranotaenia*	*damasei*	M F	X	E		P		
*Uranotaenia*	*donai*	M F	X	E		P		
*Uranotaenia*	*douceti*		X	O, W, E		P		
*Uranotaenia*	*fulgens*	M	X	E		P		
*Uranotaenia*	*grenieri*		X	C, E		P, N		
*Uranotaenia*	*grjebinei*		X	E		P		
*Uranotaenia*	*haddowi*		X	O, N, W, E		P		
*Uranotaenia*	*hervyi*		X	E		P		
*Uranotaenia*	*hirsuta*	M F	X	W		N		
*Uranotaenia*	*kraussi*		X	N, C		P		
*Uranotaenia*	*laffosseae*		X°	N, E		P		
*Uranotaenia*	*lavieri*	M F	X	O, N, E		P		
*Uranotaenia*	*legoffi*		X	E		P		
*Uranotaenia*	*longitubus*	M F	X	E		P		
*Uranotaenia*	*lousthei*	M F	X	E		P		
*Uranotaenia*	*madagascarensis*	X	E		P			
*Uranotaenia*	*manakaraensis*	L	X	E		P		
*Uranotaenia*	*nigricephala*		X	E		P		
*Uranotaenia*	*nigripleura*	L	X	E		P		
*Uranotaenia*	*pallidipleura*	L	X	E		P		
*Uranotaenia*	*pauliani*	M F	X	C		P		
*Uranotaenia*	*pilosa*	F	X	C, E		P		
*Uranotaenia*	*pseudoalbimanus*		X	E		P		
*Uranotaenia*	*pseudoshillitonis*		X	C, E		P		
*Uranotaenia*	*ravenalaphila*	M F	X	E		P		
*Uranotaenia*	*ravenalicola*		X	E		P		
*Uranotaenia*	*scutostriata*		X	E		P		
*Uranotaenia*	*spinitubus*	M F	X	E		P		
*Uranotaenia*	*spinosa*	M	X	E		P		
*Uranotaenia*	*spiraculata*		X	E		P		
*Uranotaenia*	*tricolor*	M F	X	E		P		
*Uranotaenia*	*tridentata*	M F	X	E		P		
*Uranotaenia*	*tsaratananae*		X	N, E		P		
*Uranotaenia*	*alba*			E		A		
*Uranotaenia*	*alboabdominalis*			W, E		N		
*Uranotaenia*	*albocephala*	M	X	E		P		
*Uranotaenia*	*andavakae*		X°	W, C, E		A, N		
*Uranotaenia*	*anopheloides*		X°	W, C, E		P		
*Uranotaenia*	*argentipleura*	L	X	O, W, S		N		
*Uranotaenia*	*balfouri*			O, W, C, E		A, N		
*Uranotaenia*	*bidentata*	M F	X	E				
*Uranotaenia*	*connali*	L		E				
*Uranotaenia*	*dumonti*	M F	X	C		N		
*Uranotaenia*	*geniculata*		X	E		P		
*Uranotaenia*	*grassei*	M	X	E		N		
*Uranotaenia*	*hamoni*		X	W, C, E		A, N		
*Uranotaenia*	*hebrardi*		X	W, C, E		A, N		
*Uranotaenia*	*joucouri*	M F	X	E				
*Uranotaenia*	*lebiedi*	L	X	E				
*Uranotaenia*	*madagascarica*	X	E		N			
*Uranotaenia*	*mayottensis*		X°	W, E, S		N		
*Uranotaenia*	*moramangae*		X	C, E, S		N		
*Uranotaenia*	*neireti*		X	C, E				
*Uranotaenia*	*roberti*	M F	X	E		N		

M: male, F: female, L: larva (only the information about the stages of development routinely used in taxonomy are indicated: adult and larva).

° Endemic in Madagascar and Comoros archipelagos specific bioclimatic domains, W: west, C: center, E: east, N: north, S: south, O: Sambirano (in case of presence in all climatic domains the response remained blank).

? The locality where this species was collected was not well specified.

O: ornitophile, Z: zoophile, (ruminants), A: anthropophilic, GF: general feeder (Z, O, A). A: terrestrial water accumulation associated with agricultural activity, N: natural larval habitats associated with terrestrial habitat, P: phytotelmata (bamboo, tree hole, leaf axils), T: artificial containers (small plastic receptacles, tires, drums, cans, sewage) water accumulation (temporary or not). F: filariasis, PERV: Périnet virus, RVFV: Rift Valley fever virus, WNV: West Nile virus, BABV: Babanki virus, P: *Plasmodium* parasite, NGAV: Ngari virus, ANDV: Andasibe virus, DBV: Dakar Bat virus, BTV: Bluetongue virus, MgV: Mengo virus.

£ mixed batch of mosquito species.

V: virus (arbovirus or not).

* Presence of at least an oocyst stage of *Plasmodium.* μ = avian *plasmodium.*

$ On Comoros island.

§ In Japan.

The report of the genus *Culiseta* (*Culiseta longiareolata* (Macquart)) as present in Madagascar in the Arim dataset [5] is doubtful as information on collection areas is not available. To date, there is no conclusive evidence of the presence of this tropical species in Madagascar and this information was treated as an error.

Taking into account that 3546 mosquito species are currently recognized in the world [119], 6.6% of them are present in Madagascar. In addition, 28% of the 804 known species in the Ethiopian zoogeographical area are present in Madagascar [144]. Moreover, despite decades of research, there is no doubt that new species remain to be discovered and that endemism could be higher than 60%. Indeed, some species have been reported only once (32 endemic species) and sometimes described from a single specimen with the possibility of misidentification (7 endemic species). In addition, species complexes exist, and several new species could occur without having been identified and described. On the other hand, due to deforestation, anthropogenization, and degradation of natural (and poorly explored) biotopes, several mosquito species have probably already disappeared.

### 3.2 Endemism rate of Malagasy mosquitoes

Mosquitoes already existed before separation of Madagascar and the Indian subcontinent from Africa, 156 million years ago, during the Upper Jurassic. The phylogeographic origins of sylvatic Malagasy mosquito species are not easy to decipher, and we do not yet know when and how founder species reached Madagascar. Differentiation occurred in nearly all the country’s biogeographic domains. It was not observed in the southern domain because no strictly southern endemic species is known. However, there are Malagasy endemic mosquitoes in all biogeographic domains, including the south where more than 33% of species are endemic. The eastern domain, where the relict rainforest is still present, has the highest number of mosquito species (*n* = 190) and level of regional endemism (67%). Several species are microendemics, only present (or described) in a single biotope, often in forested areas (e.g., *Ae. ambreensis*, *Ae. albodorsalis*, *Ae. mathioti*, *An. roubaudi*, *Mi. longicornis*, *Mi. mattinglyi*, *Mi. stellata*, and *Or. ankaratrensis*, all species belonging to the genus *Toxorhynchites*, and eight species belonging to the genus *Uranotaenia*).

There is no endemic genus in Madagascar. Mosquito species endemism occurs in 9 of the 13 genera of mosquito, with variation of the endemism rate from 27% to 100%, according to the genus.

The genus *Aedeomyia* has 66% (2/3) endemism. This genus is distributed in the Australasian, Oriental, African, and Neotropical regions [117]. The known species would appear to be from a primitive population that developed during the fragmentation of Gondwana 100 million years ago [33].

The genus *Aedes* (which includes the following subgenera, or genera in the new classification: *Aedimorphus*, *Coetzeemyia*, *Diceromyia*, *Fredwardsius*, *Mucidus*, *Neomelaniconion*, *Ochlerotatus*, *Skusea*, *Stegomyia*, and *Zavortinkius*) has 57% (20/35) endemism. This genus of Malagasy fauna has a higher affinity with those of the African continent, Indonesian region [182], and Oriental region [193].

The genus *Anopheles* has a cosmopolitan distribution [117]. All non-endemic *Anopheles* species are known from broad regions of Africa. The *Anopheles* fauna of Madagascar is typically Ethiopian and the occurrence of high endemism (46%; 12/26) supports evidence that its separation from the mainland must have taken place a long time ago [70].

The genus *Coquillettidia* is distributed in the Old World and Neotropical region [117]. In Madagascar, this genus has 66% (2/3) endemism.

For the genus *Culex*, the majority of non-endemic species are of African origin and only the subgenus *Culex* contains a few species occurring outside the African region (species belonging to the groups Univittatus and Pipiens). *Culex* species, endemic to Madagascar, belong to five subgenera. Today, there are less than 24% endemic *Culex* species in Madagascar and the best known bioclimatic area for this genus is probably the central domain. In contrast, no endemic *Culex* species are known in the western, northern, and southern domains. There is no doubt that endemicity should be higher in this genus. Already recorded species belong to species complexes, and a revision of genus *Culex* would likely modify the species inventory in the future.

The genus *Mimomyia* has a high endemism rate (77%; 17/22). The subgenus *Ingramia* is well represented and all species belonging to this subgenus are endemic to Madagascar. The subgenus *Etorleptiomyia* is found from Africa to the Oriental and South Pacific regions. The subgenus *Mimomyia* is widely distributed on the African mainland and extends in the east to New Guinea, north-eastern Australia, and the South Pacific [110].

The genus *Orthopodomyia* is distributed throughout the Afrotropical, Nearctic, Neotropical, Palaearctic, and Oriental regions [117]. However, all Malagasy species of *Orthopodomyia* are endemic (8/8) and belong only to the Vernoni Group. Because of their morphological homogeneity, these species are probably derived from a single ancestral species [27].

The genus *Toxorhynchites* with four subgenera is distributed throughout the Afrotropical, Australasian, Neotropical, eastern Palaearctic, and Oriental regions [117]. All Malagasy species of *Toxorhynchites* are endemic (6/6) and belong only to the subgenus *Afrorhynchus*. This subgenus evolved here after the separation of Madagascar from the African mainland during the Tertiary period [190].

The genus *Uranotaenia* is represented by *Pseudoficalbia* and *Uranotaenia* subgenera. All species of subgenus *Pseudoficalbia* are endemic to Madagascar and affinity between Malagasy, African, and Indo-Malaysian species is observed [51]. These authors suggested that the subgenus *Pseudoficalbia* seems to have appeared in the Madagascar-Indian plate boundary during the Upper Cretaceous. Most species belonging to the subgenus *Uranotaenia* exhibit a marked affinity with African and Indian species and high endemic components are observed in different sections. Specific radiation seems to have appeared before Gondwana fragmentation [51].

There are no endemic species within the genera *Eretmapodites*, *Ficalbia*, *Lutzia*, and *Mansonia*. It is important to note that these genera are represented at most by four species. The genus *Eretmapodites* occurs only in the Afrotropical region, the two *Ficalbia* species have Afrotropical origin [110]; *Lutzia* is present in the Neotropical, Asian, Australasian, and African regions and *Mansonia* has worldwide distribution [117].

### 3.3 Bio-indicator species

According to Leclercq [139], a good bio-indicator should have a specific exigency allowing a link between its presence/absence and environmental particularities. Mosquitoes fit into this definition [62].

#### 3.3.1 Urban species

Mosquito species which grow in peri- and para-domestic breeding sites are characteristic of urban areas [240]. Mosquito species growing in several artificial and polluted breeding sites exist in Malagasy urban habitats. It is not surprising to find a high abundance of *Cx. quinquefasciatus* and *Ae. albopictus* in Antananarivo, the capital and most urbanized city of Madagascar [85, 176] where discarded bottles represent 64% of household waste [178] and the rice fields of the bottom-land are converted into polluted watercress fields [9].

The same situation is observed in Toamasina, the second largest city in Madagascar, where *Cx. quinquefasciatus* and *Ae. albopictus* have colonized several peri- and para-domestic breeding sites [85]. These species are already considered an urban species in the tropical and south-east Asian regions [240] and in the Mascareignes Archipelago [208].

Ancient entomological data have suggested the presence of *Ae. aegypti* as a bio-indicator of urban areas in Madagascar [85]. This is however probably not true because its distribution is actually limited to smaller anthropized and/or forested areas [176].

However, *An. squamosus* and *An. coustani* are the most abundant *Anopheles* species in the peripheral areas of the capital city Antananarivo [85], and in the suburban areas of the eastern and central domains where rice fields are abundant in and around cities [85, 210].

A study performed in the Morondava region showed the abundance and concomitant presence of *Ma. uniformis* and *Ma. africana* in the cities of the western domain [85]. Indeed, all western plains, including within and on the outskirts of cities, are dotted with numerous swamps and ponds [36] which are typical habitats of these two species. These species constitute a major nuisance in some cities of south-east Asia and on the African mainland [240].

#### 3.3.2 Forested and rural species

In total, 78 species are restricted to the rainforest biotope and can be considered as bio-indicator of this habitat. Mosquito species belonging to *Anopheles* of the Neomyzomyia series [108, 112], *Aedeomyia* (*Aedeomyia*) *madagascarica* [33], *Aedes* (*Aedimorphus*) *albodorsalis* [86], *Aedes* (*Diceromyia*) *sylvaticus* [26], and endemic species of *Mimomyia* (*Ingramia*) [66, 110] were collected only in rainforest habitats. Unlike other follow-up studies [85, 227], *Cx. pipiens* was abundant only in the Anorana rainforest in the central domain [210]. Because of its abundance, *Cx. pipiens* could be considered as a bio-indicator of the rainforest habitat in the central domain. This finding raises important questions regarding the differentiation history or behavior modification of *Cx. pipiens* in relation to urban and suburban habitats [123, 180, 227]. Little information is available about its biology in Madagascar, even though a much lower proportion of this species was reported in the Andasibe rainforest of the eastern domain [85].

Several studies have reported that mosquito species of *Ficalbia*, *Toxorhynchites*, *Mimomyia* (*Ingramia*), and large numbers of *Uranotaenia* genera (especially subgenus *Pseudoficalbia*) occur only in the eastern region. These species lay their eggs in phytothelm breeding sites (*Typhonodorum*, *Ravenala*, *Pandanus*, and *Nepenthes madagascarensis*) which are characteristic of the eastern domain [35, 122, 136]. These observations confirm that these mosquito genera could be considered as good indicators of the eastern domain.

*Eretmapodites quinquevittatus* is considered as indicator species of degraded areas in all domains, with the exception of the central domain where this species is rare [85]. Four species *Aedes brygooi*, *Ae. coulangesi*, *Ae. albiradius*, and *Ae. aegypti* are confined to the driest forest of the western domain. In this domain, *Ae. fryeri* and *Ae. cartroni* species seem to be related to the presence of mangrove [182]. *Anopheles merus* was also found in dry regions in the western domain and far south-east of Madagascar and is probably associated with salt-water.

Few species are strongly represented in the warmer and dryer areas of the western domain, even though they occur in others domains: *Ae. coulangesi*, *Ae. durbanensis*, *Ae. brygooi*, *Ae. albocephalus*, *Ae. tiptoni*, *Ae. cartroni*, *Cx. tritaeniorhynchus*, and *Ur. anopheloides* are abundant in this area [31, 85, 182, 194]. Similarly, *Ae. scatophagoides* is present only in the semi-arid Androy region, in the southern domain [182], while *Ae. ambreensis* occurs only in the northern domain [85]. These observations allow us to consider these species bio-indicators of their corresponding geographical domains.

### 3.4 Mountain species

Few species have been caught in mountainous areas, at 1500 m asl, and could be considered as characteristic of this biotope. These species are *Uranotaenia andavakae* [65], *Ur*. *dumonti* [64], *Ur. hamoni* [112], *Ur*. *pauliani* [64], *An. griveaudi* [106], and *Orthopodomyia ankaratrensis* [27]. Of these, only four species are strictly endemic to high mountains (the Ankaratra and Ambohiby mountains near Tsiroanomandidy).

## 4. Conclusion

In Madagascar, up to February 2016, 235 mosquito species belonging to 14 genera have been reported, with a high level of endemism. There is no doubt that this inventory will change with new species. The taxonomic status of species complexes described from only one specimen or from one stage should be deciphered. Further description of species belonging especially to the genus *Hodgesia*, and the subgenus *Culex* (*Eumelanomyia*), should be carried out to complete this inventory.
